# Integration of Transcriptomic and Metabolomic Profiling as a Source of Insight into Key Candidate Genes and Metabolic Pathways Associated with Maize Resistance to *Fusarium* spp.

**DOI:** 10.3390/ijms27219813

**Published:** 2026-11-01

**Authors:** Maciej Lenort, Agnieszka Tomkowiak, Jan Bocianowski, Kinga Stuper-Szablewska, Tomasz Kosiada, Karolina Jarzyniak, Roksana Bobrowska, Łukasz Wolko, Przemysław Gawrysiak, Wiktoria Padiasek

**Affiliations:** 1 Department of Genetics and Plant Breeding, Poznań University of Life Sciences, Dojazd 11, 60-632 Poznań, Poland; maciej.lenort@up.poznan.pl (M.L.); roksana.bobrowska@up.poznan.pl (R.B.); przemyslaw.gawrysiak@up.poznan.pl (P.G.); wiktoriapadiasek07@gmail.com (W.P.); 2 Department of Mathematical and Statistical Methods, Poznań University of Life Sciences, Wojska Polskiego 28, 60-637 Poznań, Poland; jan.bocianowski@up.poznan.pl; 3 Department of Chemistry, Faculty of Forestry and Wood Technology, Poznań University of Life Sciences, Wojska Polskiego 28, 60-637 Poznań, Poland; kinga.stuper@up.poznan.pl; 4 Department of Phytopathology, Seed Science and Technology, Poznań University of Life Sciences, Dąbrowskiego 159, 60-594 Poznań, Poland; tomasz.kosiada@up.poznan.pl; 5 Department of Biochemistry and Biotechnology, Poznań University of Life Sciences, Dojazd 11, 60-632 Poznań, Poland; lukasz.wolko@up.poznan.pl

**Keywords:** *Zea mays*, *Fusarium* spp., *Fusarium* resistance, gene expression, metabolomics, phenolic acids, flavonoids, carotenoids, oxidoreductases, marker-assisted selection

## Abstract

*Fusarium* spp. infections substantially reduce maize productivity, making the identification of molecular and metabolic mechanisms underlying host resistance important for breeding. This study aimed to characterize the maize response to *Fusarium* infection by integrating pathogen colonization, the expression of 17 candidate genes, and metabolite profiles. Five maize genotypes differing in resistance were inoculated with a mixture of *Fusarium* spores and analyzed at 0, 6, 12, 24, and 48 h post-inoculation. Six *Fusarium* species were identified, with *F. poae* and *F. culmorum* predominating in susceptible genotypes. Colonization exceeded 70% in susceptible samples, whereas resistant genotypes generally showed 0–2% colonization. Gene expression patterns were strongly genotype- and time-dependent, with 24 h post-inoculation representing a major point of transcriptional activation. Several genes were strongly induced in the susceptible genotype, whereas selected genes, including *Putrescine hydroxycinnamoyltransferase* (LOC103649226), showed constitutive or induced expression associated with resistant genotypes. Resistant lines, particularly SMH 57524 and SMH 57624, showed higher concentrations of selected phenolic acids and luteolin glycosides and higher peroxidase, polyphenol oxidase, and catalase activities. Regression analyses revealed significant associations between candidate gene expression and the accumulation of selected metabolites, including ferulic, diferulic, p-coumaric, and caffeic acids, as well as lutein, with both positive and negative relationships being detected. These findings indicate that maize resistance to *Fusarium* is associated with coordinated transcriptional, metabolic, and redox responses and identify gene–metabolite relationships relevant to the molecular characterization of resistance.

## 1. Introduction

Maize (*Zea mays* L.) is a fundamental pillar of global food security and the economic stability of the agri-food sector [1]. As a key species in modern agriculture, it is a dominant cereal crop that provides essential raw materials for human nutrition, animal feed production, and the rapidly growing bioenergy and biopolymers sector [2,3]. The economic importance of maize cultivation on a European and global scale is underlined by its status as the species with the highest grain production volume, exceeding one billion tonnes annually [4,5].

Nevertheless, climatic disturbances significantly shape the contemporary agricultural landscape. Global warming, characterized by higher average temperatures and reduced rainfall, creates environmental conditions conducive to escalating threats to maize crops. These phenomena have not only forced the expansion of cultivated areas but have also triggered the spread of a range of pests, leading to fundamental changes in plant–pathogen interactions [6,7].

Among the biotic factors limiting maize productivity, infections caused by fungi of the genus *Fusarium* (in *particular F. poae*, *F. culmorum*, and *F. verticillioides*, which occur with high frequency in central and western Poland). Beyond quantifiable yield losses, *Fusarium* species pose a serious quality threat because they biosynthesize secondary metabolites known as mycotoxins (e.g., fumonisins, deoxynivalenol, zeara-lenone). *F. poae*, has been described as one of the most prominent *Fusarium* head blight (FHB) species in several studies [8,9]. Its occurrence is often correlated with the incidence of other *Fusarium* species and its preponderance increased with the application of azole fungicides. In turn *Fusarium culmorum* (W.G. Smith) Sacc. is a ubiquitous soil-borne fungus with a highly competitive saprophytic capability. As a facultative parasite, it is able to cause foot and root rot (FRR) and *Fusarium* head blight (FHB). Yield and quality losses are particularly important when *F. culmorum* induces FHB, which develops from infection at anthesis and spreads until grain harvest, causing grain contamination with mycotoxins, such as type B trichothecenes and zearalenone [10]. In turn, fumonisins are mycotoxins (MTs) produced mainly by the fungus *Fusarium verticillioides*, the main pathogens of maize which cause ear rot. The current tolerance limit for fumonisin is 1.5 μg g^−1^. High severity of grains infected with *F. verticillioides* does not always show high concentrations of fumonisins. Environments with higher temperatures may influence the production of high concentrations of fumonisin in maize hybrids [11,12,13]. These compounds, which accumulate in grains, pose significant health risks to humans and farm animals due to their chronic, acute, teratogenic, and mutagenic properties [14,15]. Reducing mycotoxin contamination is a public health priority because these metabolites can disrupt hormonal pathways and impair the immune response [16].

The management of *Fusarium* diseases is constrained by the lack of chemical plant protection products that ensure fully effective control. Consequently, modern integrated pest management strategies emphasize genetically based resistance as a more sustainable and environmentally friendly alternative to synthetic fungicides [16,17].

This evolution is further driven by stringent European and global regulatory frameworks, which call for reduced chemical inputs to support safer, more environmentally friendly agricultural production systems [18]. The utilization of natural host defense mechanisms is therefore a primary objective of modern breeding programs [19,20].

To accelerate the development of resistant varieties, it is essential to use advanced molecular tools that enable precise identification of candidate genes and metabolic pathways involved in the plant’s defense response [21,22]. High-throughput transcriptomic profiling and association mapping studies have become key tools in deciphering the complex, polygenic nature of *Fusarium* resistance. Extensive QTL mapping studies using various maize populations have been conducted over recent decades, identifying resistance QTLs across all ten maize chromosomes. Transcriptomic studies have further enabled the discovery of key functional genes underlying FER resistance. For instance, *ZmMPK3* was identified as a core resistance gene, while the loss of *ZmWRKY125* function was shown to enhance resistance through the activation of jasmonic acid and abscisic acid signaling pathways. Furthermore, *ZmSIZ1a* and *ZmSIZ1b* were found to mediate resistance by modulating early defense responses and MAPK signaling pathways [23]. However, the molecular mechanisms underlying FER resistance remain largely unclear, and more effective strategies are needed to identify key defense pathways and resistance-related genes. Transcriptome analysis serves as a powerful tool for identifying potential resistance genes by examining differential expression across genotypes and time points, and it is widely used to analyze disease resistance mechanisms. Metabolomics, acting as a crucial complement to transcriptomic research, provides an essential approach for identifying potential resistance-related metabolites. With the rapid development of omics technologies, integrated transcriptomic and metabolic analyses have become effective strategies for analyzing complex regulatory networks and identifying key genes and metabolites involved in plant defense responses [24].

This paper presents results from a study analyzing the expression of 17 candidate genes, systematically selected by the team for their potential role in mechanisms underlying maize defense against fungi of the genus *Fusarium*. A comprehensive understanding of their functional characteristics is essential, as modulation of their expression patterns directly can determines the plant’s physiological and biochemical response to pathogen colonization [25,26].

However, transcriptomic data alone provide only a partial view of the molecular processes underlying plant defense responses. Consequently, the team carried out a complementary analysis of metabolomic profiles, which, according to other authors [27,28], can provide valuable insight [29]. Metabolites may function as constitutive or induced compounds, acting directly on the pathogen or serving as signaling molecules that regulate systemic immunity [30].

In this study, we hypothesize that the observed variability in gene expression levels translates into corresponding fluctuations in key metabolite concentrations, bridging the genotype and the resulting defense phenotype [31,32].

This publication aims to provide a comprehensive molecular and metabolic characterization of maize (*Zea mays* L.) resistance to infections caused by fungi of the genus *Fusarium*, with particular emphasis on interactions between the host transcriptome and metabolome [33].

As part of the implementation of this main objective, the following specific objectives were also achieved: comparative analyses of gene expression patterns were conducted (to demonstrate differences in the dynamics of the transcriptional response between genotypes with varying susceptibility to the pathogen), metabolic biomarkers were identified (to determine the profile of secondary metabolites correlated with the activity of defense genes, in order to identify potential chemical indicators of resistance), omics data were integrated (to elucidate the functional relationships between selected genes and the accumulation of specific chemical compounds, which will enable the interpretation of the metabolic pathways involved in limiting *Fusarium* colonization), and results were validated (to assess the suitability of the identified molecular markers for optimizing marker-assisted selection—MAS—programs, in order to accelerate the breeding of maize varieties with enhanced field resistance). By integrating omics data, this work aims to expand knowledge of the architecture of plant resistance and provide tools to support sustainable crop protection strategies in the face of intensifying climate change.

## 2. Results

### 2.1. Identification of Fungal Species of the Genus Fusarium

**Figure 1:**
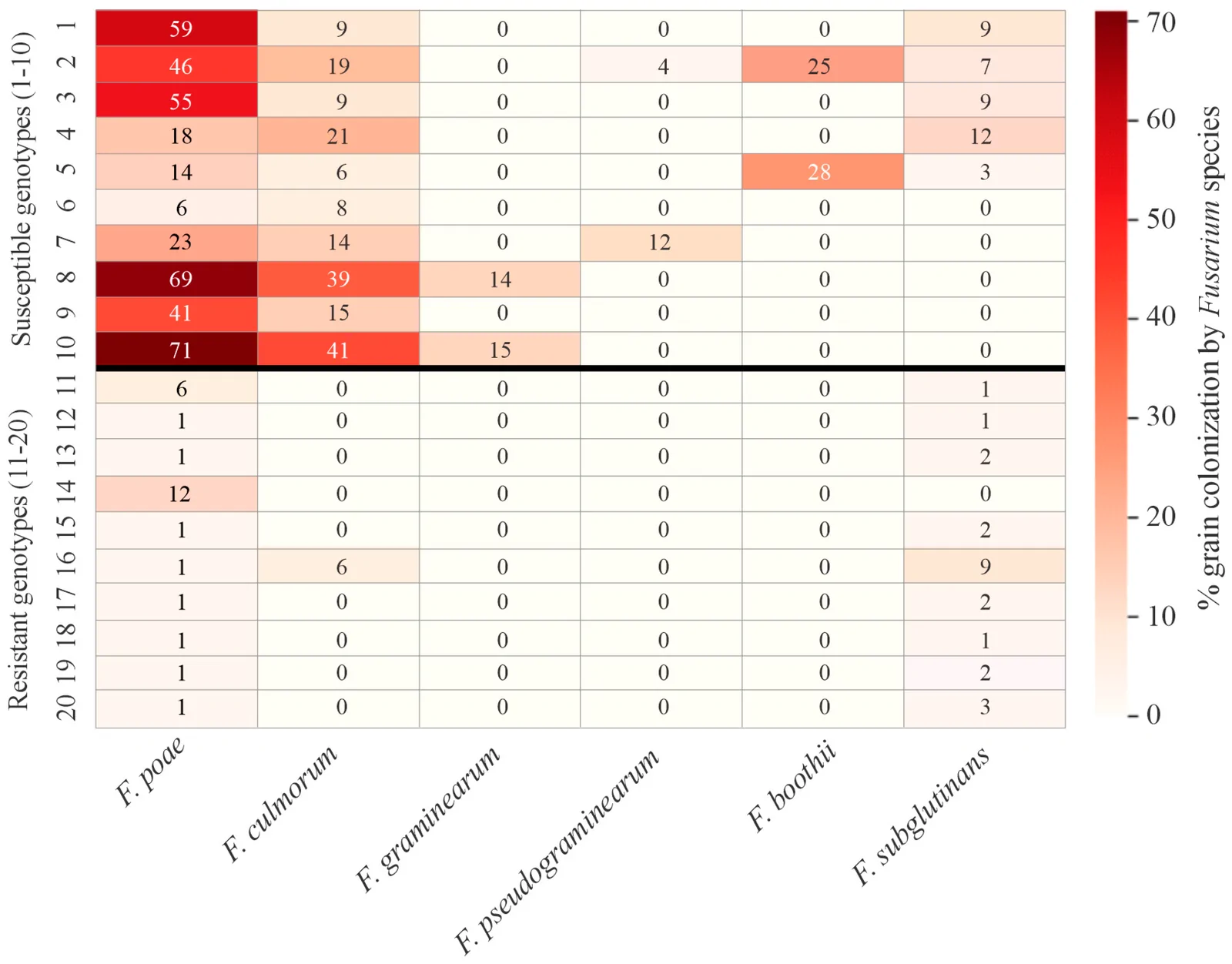
Heatmap of maize grain colonization by *Fusarium* species.

The heatmap (Figure 1) illustrates the degree of colonization of maize grains, expressed as a percentage, by six selected *Fusarium* species: *F. poae*, *F. culmorum*, *F. graminearum*, *F. pseudograminearum*, *F. boothii*, and *F. subglutinans*. The analysis covers 20 maize genotype samples, divided into two experimental groups along the horizontal axis: susceptible genotypes (1–10) and resistant genotypes (11–20). The analysis revealed a clear dominance of *F. poae* and *F. culmorum* during grain colonization, particularly among the group of susceptible genotypes. In particular, *F. poae* achieved the highest colonization rates, reaching 71% (sample 10) and 69% (sample 8). In the susceptible genotypes (1–10), infection was high, mainly due to *F. poae* (typically above 18%, and in many cases exceeding 40–70%) and *F. culmorum* (reaching 41%). In the group of resistant genotypes (11–20), the level of colonization by all analyzed *Fusarium* species drops dramatically, reaching marginal values in the range of 0–2% (with the exception of sample 14, which showed 12% colonization by *F. poae*, and isolated instances of low values for *F. subglutinans* and *F. poae*). The results obtained confirm a marked variation in the susceptibility of the tested maize genotypes to infection by specific species of the *Fusarium* complex. A clear correlation was demonstrated between genotypes belonging to the susceptible group and high colonization intensity by *F. poae* (*t*_9.43_ = 4.97, *p* < 0.001) and *F. culmorum* (*t*_9.41_ = 4.37, *p* = 0.002), whereas resistant genotypes exhibited a strong ability to restrict the development of all monitored pathogens.

### 2.2. Analysis of the Transcriptional Activity of Candidate Genes

#### 2.2.1. Analysis of the Expression of the Candidate Gene *GDSL Esterase/Lipase At4g01130 LOC100273960*

**Figure 2:**
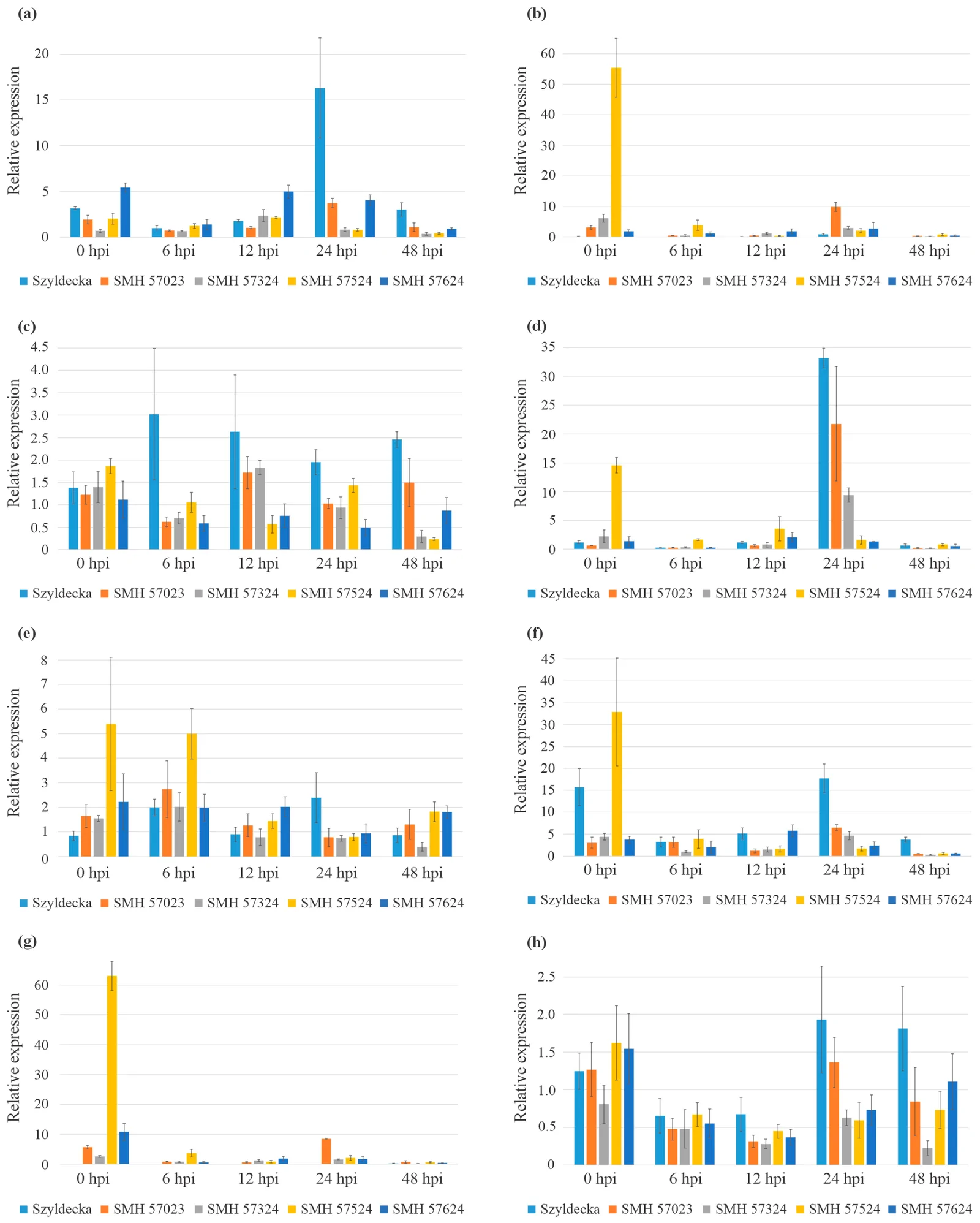
Graphs showing the transcriptional activity of candidate genes: (**a**) Gene 1—*LOC100273960*, (**b**) Gene 2—*LOC103649226*, (**c**) Gene 3—*LOC100275570*, (**d**) Gene 4—*LOC100383808*, (**e**) Gene 5—*LOC100285378*, (**f**) Gene 6—*LOC100285600*, (**g**) Gene 7—*LOC100274850*, (**h**) Gene 8—*LOC100278673* associated with maize resistance to fungi of the genus *Fusarium*. Least significant difference (LSD) values: Gene 1: Cultivar: 1.48; Time: 1.48; Cultivar × Time: 3.309; Gene 2: Cultivar: 2.752; Time: 2.752; Cultivar × Time: 6.153; Gene 3: Cultivar: 0.5818; Time: 0.5818; Cultivar × Time: 1.301; Gene 4: Cultivar: 2.698; Time: 2.698; Cultivar × Time: 6.034; Gene 5: Cultivar: 0.976; Time: 0.976; Cultivar × Time: 2.183; Gene 6: Cultivar: 3.582; Time: 3.582; Cultivar × Time: 8.009; Gene 7: Cultivar: 1.697; Time: 1.697; Cultivar × Time: 3.794; Gene 8: Cultivar: 0.3981; Time: 0.3981; Cultivar × Time: 0.8902.

The transcriptional profile of gene 1 (*LOC100273960*) (Figure 2a), which encodes a GDSL esterase/lipase protein, revealed a marked contrast between the susceptible cultivar Szyldecka and the resistant maize lines following *Fusarium* inoculation. Basal transcript levels varied among genotypes, with the highest pre-inoculation expression detected in the resistant line SMH 57624 (approximately 5.5). A rapid decline in expression was observed across all genotypes at 6 hpi, which may indicate a common early response to pathogen challenge or the circadian rhythm, since the six-hour mark post-inoculation coincided with midnight. It is possible that, in the absence of light, the transcription of genes related to metabolism or photosynthesis underwent a physiological downregulation, which could explain the uniform decrease in G1 (*LOC100273960*) gene expression across all genotypes. Thereafter, genotype-specific expression patterns became apparent. The most prominent feature was a strong and transient induction in the susceptible cultivar Szyldecka at 24 hpi, where transcript abundance increased several-fold relative to earlier time points. In contrast, the resistant lines displayed only moderate fluctuations in expression and lacked a comparable increase in transcript accumulation. The higher expression of the G1 gene in the susceptible Szyldecka variety compared to resistant lines at 24 hpi (hours post-inoculation) may stem from differences in the mechanisms and dynamics of the defense response between susceptible and resistant genotypes. Resistant lines (SMH 57023, SMH 57324, SMH 57524, SMH 57624) may possess more efficient pathogen-recognition mechanisms (e.g., PRR/NLR receptors) and pre-existing constitutive barriers or very early responses (e.g., an ROS burst); consequently, they do not need to maintain or induce drastically high levels of G1 gene expression. In contrast, for the susceptible Szyldecka variety, the high G1 gene expression at 24 hpi may signal severe infection stress and an attempted defense response in tissue already heavily colonized by the pathogen. By 48 hpi, transcript levels had declined in all genotypes, approaching their initial values.

#### 2.2.2. Analysis of the Expression of the Candidate Gene *Putrescine Hydroxycinnamoyltransferase LOC103649226*

Unlike gene 1 (*LOC100273960*), the expression profile of gene 2 (*LOC103649226*) (Figure 2b), which encodes the putrescine hydroxycinnamoyltransferase protein, showed strong genotype-dependent regulation largely restricted to resistant maize lines. The most notable feature was the exceptionally high constitutive expression in the resistant line SMH 57524 (yellow bar) before inoculation, averaging approximately 55. SMH 57324 and SMH 57023 showed lower but still detectable basal expression (approximately 6 and 3, respectively). In contrast, the susceptible cultivar Szyldecka exhibited near-background transcript levels and remained among the lowest-expressing genotypes throughout the experiment. Following inoculation, the elevated expression detected in SMH 57524 declined rapidly. At 24 hpi, however, a distinct genotype-specific induction emerged in another resistant line, SMH 57023 (orange bar), where transcript abundance increased to approximately 10, while the remaining genotypes maintained minimal expression. By 48 hpi, transcript accumulation had returned to near-background levels in all lines.

The restriction of both constitutive and inducible expression to resistant genotypes, together with the absence of a measurable response in Szyldecka, suggests that Putrescine hydroxycinnamoyltransferase may be associated with resistance-related responses operating in specific genetic backgrounds rather than with a general transcriptional response to *Fusarium* infection.

#### 2.2.3. Analysis of the Expression of the Candidate Gene Encoding the Uncharacterized Protein LOC100275570

Gene 3 (*LOC100275570*), encoding an uncharacterized protein, displayed a distinctive expression pattern (Figure 2c) characterized by rapid activation in the susceptible cultivar Szyldecka following *Fusarium* inoculation. Prior to infection, transcript levels were low and relatively similar across all genotypes. However, by 6 hpi, Szyldecka displayed a marked increase in transcript abundance (approximately 3.0), whereas all resistant lines showed reduced expression relative to their initial levels. This divergence persisted throughout the infection period. Transcript abundance in Szyldecka remained elevated during the later stages of infection, ranging from approximately 1.9 to 2.6 between 12 and 48 hpi. In contrast, expression in the resistant lines remained comparatively low. Although transient increases were observed in SMH 57023 and SMH 57324 at 12 hpi, these responses were not sustained and declined at subsequent time points.

The early onset of transcriptional activation and its preferential accumulation in the susceptible genotype distinguish LOC100275570 from most other candidate genes analyzed in this study. The strong association between elevated transcript levels and the susceptible phenotype suggests that this gene may represent a putative marker of susceptibility-related responses to *Fusarium* infection.

#### 2.2.4. Analysis of the Expression of the Candidate Gene Encoding the Uncharacterized Protein LOC100383808

Among the candidate genes analyzed, *LOC100383808* (Gene 4, Figure 2d) showed a narrow but remarkably intense expression peak at 24 hpi. Transcript accumulation increased dramatically in the susceptible cultivar Szyldecka, reaching approximately 33, and, to a lesser extent, in the resistant lines SMH 57023 and SMH 57324. This response was transient, as expression returned to near-background levels by 48 hpi. In contrast to the pronounced activation observed at 24 hpi, transcript levels were generally low before inoculation and during the early stages of infection. The only exception was the resistant line SMH 57524, which displayed elevated basal expression (approximately 14.6) relative to the other genotypes. However, this initially high transcript abundance declined rapidly following inoculation and was not maintained during subsequent stages of infection.

The transient activation observed at 24 hpi in both susceptible and resistant genetic backgrounds suggests that *LOC100383808* forms part of a broader transcriptional response to *Fusarium* infection rather than being specifically associated with either resistance or susceptibility. Nevertheless, the markedly stronger induction in Szyldecka indicates that the contribution of this gene may differ among genotypes.

#### 2.2.5. Analysis of the Expression of the Candidate Gene Encoding the Uncharacterized Protein LOC100285378

Gene 5 (*LOC100285378*) showed a dynamic expression profile (Figure 2e), with the highest transcript levels shifting from resistant genotypes before inoculation to the susceptible cultivar during infection. Before inoculation, transcript abundance was highest in the resistant line SMH 57524, whereas the susceptible cultivar Szyldecka exhibited the lowest expression level (0.8) among all genotypes. This trend persisted at 6 hpi, with SMH 57524 maintaining the highest transcript accumulation (approximately 5.0) and SMH 57023 reaching a local maximum of approximately 2.7. By 24 hpi, however, expression patterns had diverged. Transcript levels in the resistant lines declined to below 1.0, while Szyldecka showed a moderate increase in expression to approximately 2.4, becoming the genotype with the highest transcript abundance at this time point. These differences were transient, and expression in all genotypes returned to levels close to those observed before inoculation by 48 hpi.

#### 2.2.6. Analysis of the Expression of the Candidate Gene Encoding the Uncharacterized Protein LOC100285600

Gene 6 (*LOC100285600,* Figure 2f), encoding an uncharacterized protein, was distinguished by high transcript accumulation in the susceptible cultivar Szyldecka both before inoculation and at 24 hpi. Prior to pathogen exposure, Szyldecka exhibited an expression level of approximately 15.7, comparable only to the resistant line SMH 57524, whereas the remaining resistant lines showed considerably lower transcript abundance (approximately 3.1–4.5). The high basal expression observed in SMH 57524 declined rapidly after inoculation and remained low throughout the experiment. In contrast, transcript accumulation increased at 24 hpi, particularly in Szyldecka (approximately 18), while SMH 57023 and SMH 57324 showed a more modest increase. Notably, the expression peak detected in Szyldecka at 24 hpi is consistent with the profiles of Genes 1 (*LOC100273960*), 3 (*LOC100275570*), 4 (*LOC100383808*), and 5 (*LOC100285378*), which exhibited their highest or preferential transcript accumulation in the susceptible cultivar at this stage of infection. Nevertheless, this response was transient, and mRNA of the analyzed gene declined in all genotypes by 48 hpi, reflecting the patterns observed for Genes 1, 2 (*LOC103649226*), and 4.

Together, these findings suggest a recurring susceptibility-associated transcriptional response that is strongest at 24 hpi but not maintained during the later stages of infection.

#### 2.2.7. Analysis of the Expression of the Candidate Gene Encoding the Uncharacterized Protein LOC100274850

Gene 7 (*LOC100274850*, Figure 2g) shared several characteristics with the expression profile observed for Gene 2 (*LOC103649226*). Both genes combined high constitutive transcript accumulation in the resistant line SMH 57524, with a transient increase in expression in SMH 57023 following inoculation. Before pathogen treatment, mRNA accumulation was highest in SMH 57524 (approximately 63), followed by SMH 57624 (approximately 11) and SMH 57023 (approximately 6), whereas expression in the susceptible cultivar Szyldecka remained near background levels. Following inoculation, transcript levels decreased across all genotypes and remained low throughout the infection period. Except for SMH 57023, which reached its maximum transcript abundance at 24 hpi (approximately 8.5).

As in Gene 2, both high constitutive expression and post-inoculation activation of gene 7 were largely confined to resistant germplasm, in contrast to the limited response observed in the susceptible cultivar.

#### 2.2.8. Analysis of the Expression of the Candidate Gene Encoding the Uncharacterized Protein LOC100278673

Compared with several previously analyzed genes, *LOC100278673* (Figure 2h), which encodes an uncharacterized protein, showed relatively moderate temporal variation in expression. Under control conditions, mRNA levels were comparable across all genotypes, ranging from approximately 0.8 to 1.6, with the highest value in the resistant line SMH 57524. Transcript abundance decreased in all genotypes during the early stages of infection, reaching its lowest levels at 6 and 12 hpi. A subsequent increase was observed at 24 hpi, with the highest expression detected in the susceptible cultivar Szyldecka (approximately 1.9), followed by SMH 57023 (approximately 1.4). Unlike the transient expression peaks observed for several previously described genes, elevated transcript levels persisted in Szyldecka until 48 hpi, when expression remained close to 1.8. A similar, although less pronounced, trend was observed in SMH 57624 (approx. 1.1), whereas expression in the remaining resistant lines declined below 0.9.

The sustained transcript accumulation observed in Szyldecka during the later stages of infection resembles the pattern previously described for Gene 3 (*LOC100275570*) and further supports an association of *LOC100278673* expression with susceptibility-related responses following *Fusarium* inoculation.

#### 2.2.9. Analysis of the Expression of the Candidate Gene Encoding the Uncharacterized Protein LOC100383775

**Figure 3:**
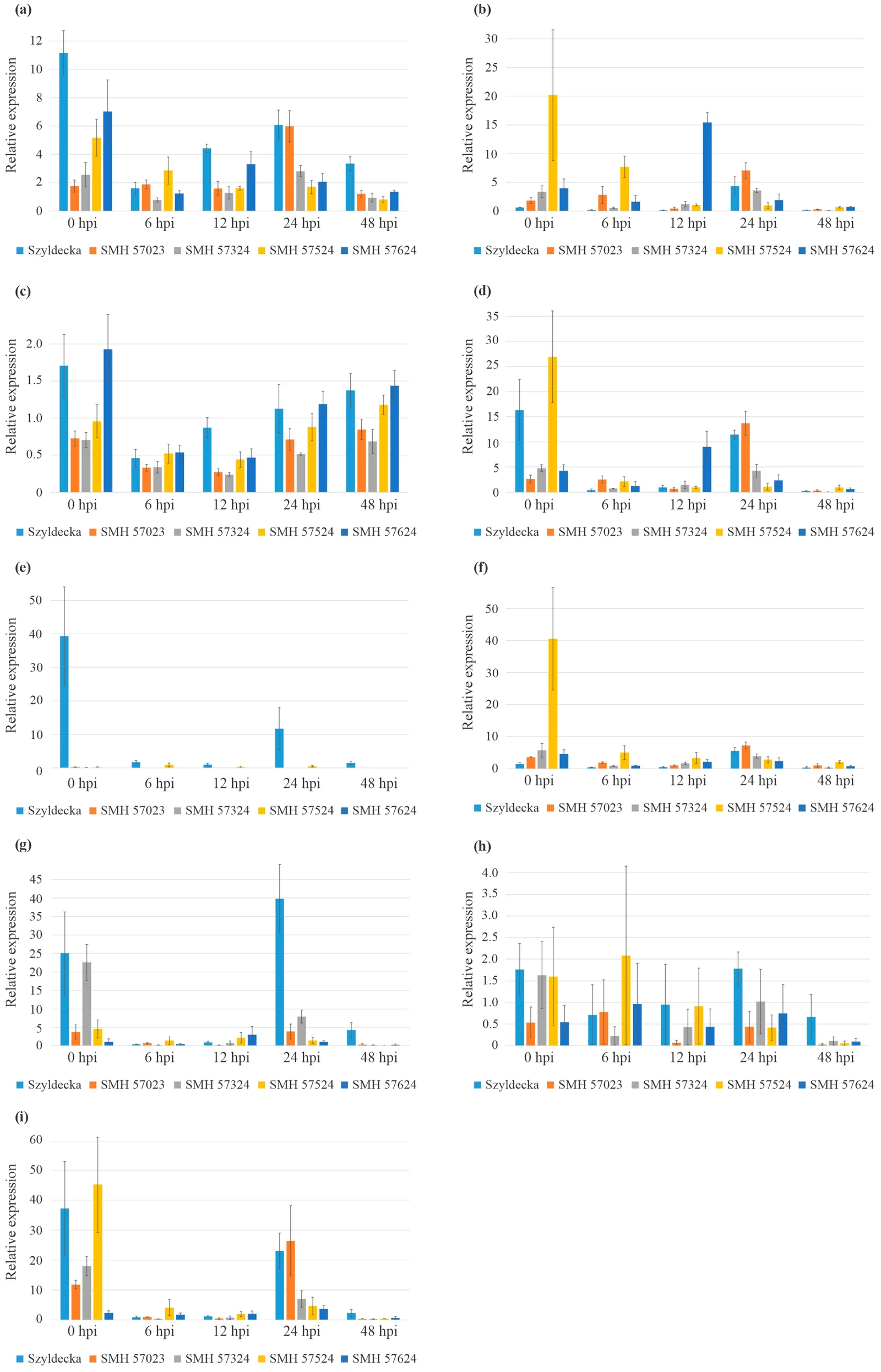
Graphs showing the transcriptional activity of candidate genes: (**a**) Gene 9—*LOC100383775*, (**b**) Gene 10—*LOC109942573*, (**c**) Gene 11—*LOC103644288*, (**d**) Gene 12—*LOC100277697*, (**e**) Gene 13—*LOC100282707*, (**f**) Gene 14—*LOC100280235*, (**g**) Gene 15—*LOC100282644*, (**h**) Gene 16—*LOC103652964*, (**i**) Gene 17—*LOC103633003* associated with maize resistance to fungi of the genus *Fusarium*. Least significant difference (LSD) values: Gene 9: Cultivar: 1.023; Time: 1.023; Cultivar × Time: 2.287; Gene 10: Cultivar: 3.111; Time: 3.111; Cultivar × Time: 6.955; Gene 11: Cultivar: 0.2419; Time: 0.2419; Cultivar × Time: 0.5408; Gene 12: Cultivar: 3.065; Time: 3.065; Cultivar × Time: 6.854; Gene 13: Cultivar: 5.545; Time: 5.545; Cultivar × Time: 12.399; Gene 14: Cultivar: 4.219; Time: 4.219; Cultivar × Time: 9.435; Gene 15: Cultivar: 4.152; Time: 4.152; Cultivar × Time: 9.283; Gene 16: Cultivar: 0.895; Time: 0.895; Cultivar × Time: 2.001; Gene 17: Cultivar: 6.84; Time: 6.84; Cultivar × Time: 15.29.

Gene 9 (*LOC100383775*, Figure 3a) displayed marked genotype-dependent differences in transcriptional regulation. Before inoculation, the susceptible genotype Szyldecka showed the highest transcript abundance, with comparatively high basal expression also observed in resistant lines SMH 57624 and SMH 57524. In contrast, SMH 57023 and SMH 57324 showed substantially lower initial transcript levels (below 2.6). After inoculation, expression decreased sharply in all genotypes, most notably in Szyldecka, substantially reducing the differences observed at 0 h. Subsequently, the analyzed genotypes showed distinct temporal responses. In Szyldecka, transcript abundance gradually increased between 6 and 24 hpi, although expression remained below the pre-inoculation level. In contrast, SMH 57023 maintained relatively stable transcript levels during the early stages of infection and displayed a pronounced induction only at 24 hpi. At this time point, both genotypes reached comparable expression levels. Expression declined again in all genotypes by 48 hpi.

This pattern suggests that similar transcript abundance at 24 hpi may arise from distinct regulatory mechanisms in susceptible and resistant genetic backgrounds. Szyldecka showed high basal expression followed by partial recovery after early repression, whereas SMH 57023 exhibited a predominantly inducible response associated with pathogen challenge.

#### 2.2.10. Analysis of the Expression of the Candidate Gene Encoding the Uncharacterized Protein LOC109942573

One of the most notable features of Gene 10 (*LOC109942573*, Figure 3b) was the contrasting temporal patterns observed among the resistant lines. Under control conditions, SMH 57524 exhibited substantially higher transcript levels (~20) than all other genotypes. This elevated basal activity was rapidly suppressed following inoculation, resulting in a progressive decline throughout the experiment. In contrast, SMH 57624 reached a pronounced transcriptional maximum at 12 hpi (approximately 15.5), exceeding levels in all other genotypes after inoculation. A different pattern emerged in SMH 57023, where transcription remained relatively stable during the early stages of infection before increasing markedly at 24 hpi (approx. 7.1). More moderate increases at the same time point were also detected in Szyldecka and SMH 57324. The transient nature of these responses became evident at 48 hpi, when transcript levels returned to low values across all genotypes.

The differences in the timing of maximal activity suggest that Gene 10 (*LOC109942573*) is regulated in a genotype-specific manner. Rather than being associated with a single resistance-related profile, this gene appears to be integrated into multiple transcriptional programs operating in different resistant backgrounds during *Fusarium* infection.

#### 2.2.11. Analysis of the Expression of the Candidate Gene Encoding Cysteine-tRNA Ligase CPS1, Chloroplastic/Mitochondrial-like Protein LOC103644288

Unlike many previously analyzed candidate genes, Gene 11 (*LOC103644288*), encoding cysteine-tRNA ligase CPS1, chloroplastic/mitochondrial-like protein (Figure 3c), did not show a pronounced transcriptional peak at 24 hpi. Instead, all genotypes exhibited a common pattern characterized by an early decline followed by a gradual recovery during the later stages of infection. Throughout the experiment, Szyldecka and SMH 57624 consistently maintained the highest transcript levels, despite experiencing the strongest initial reduction after inoculation.

In contrast to the transient activation observed for several previously analyzed candidates, the expression dynamics of Gene 11 remained relatively uniform across susceptible and resistant backgrounds. This conserved response pattern suggests that Gene 11 is more likely involved in general physiological adaptation to pathogen challenge than in genotype-specific resistance or susceptibility mechanisms.

#### 2.2.12. Analysis of the Expression of the Candidate Gene Encoding the Uncharacterized Protein LOC100277697

The expression profile of *LOC100277697* (Gene 12, Figure 3d) showed marked temporal variation among the examined genotypes. Before inoculation, SMH 57524 exhibited the highest transcript abundance (approximately 26.9), followed by Szyldecka (approximately 16.3), whereas expression in SMH 57324, SMH 57624, and SMH 57023 remained substantially lower. This initial predominance was rapidly lost following inoculation. By 6 hpi, transcript levels had declined sharply in SMH 57524 and Szyldecka, while SMH 57023 showed the smallest reduction and remained close to its initial level. At 12 hpi, SMH 57624 displayed a transient increase to approximately 9.0, exceeding all other genotypes. A second phase of elevated expression occurred at 24 hpi and was dominated by SMH 57023 and Szyldecka, which reached approximately 13.6 and 11.5, respectively. Expression in SMH 57324 increased more moderately, whereas SMH 57524 and SMH 57624 remained at relatively low levels. By 48 hpi, transcript abundance had decreased across all genotypes.

Overall, *LOC100277697* combined high constitutive expression with rapid post-inoculation suppression and a subsequent reactivation phase involving different genotypes at successive time points. The transient peak in SMH 57624 at 12 hpi, followed by elevated expression in SMH 57023 and Szyldecka at 24 hpi, highlights the strongly temporal and genotype-dependent nature of its transcriptional response.

#### 2.2.13. Analysis of the Expression of the Candidate Gene Phosphoribosylaminoimidazole-Succinocarboxamide Synthase LOC100282707

Gene 13 (*LOC100282707*), encoding phosphoribosylaminoimidazole-succinocarboxamide synthase (Figure 3e), was distinguished by an exceptionally strong genotype-specific expression pattern. Prior to inoculation, transcript levels were markedly elevated in the susceptible genotype Szyldecka (approx. 39.4), whereas all resistant lines maintained negligible expression throughout the experiment (below 0.5). Following pathogen challenge, the abundant transcript pool present in Szyldecka was rapidly depleted, resulting in a pronounced reduction in expression during the early stages of infection. Despite this decline, Szyldecka remained the only genotype displaying substantial transcript accumulation, and a secondary increase was observed at 24 hpi. In contrast, expression in all resistant lines remained consistently close to background levels across the entire time course.

The strong predominance of Gene 13 (*LOC100282707*) activity in Szyldecka, both before and after inoculation, distinguishes this gene from most other candidates analyzed in this study. The absence of a comparable response in resistant backgrounds suggests that its regulation is closely associated with the susceptible genotype and may reflect physiological processes accompanying successful pathogen establishment rather than defense-associated mechanisms.

#### 2.2.14. Analysis of the Expression of the Candidate Gene Encoding the Uncharacterized Protein LOC100280235

Gene 14 (*LOC100280235*) displayed an expression pattern (Figure 3f) partially resembling that of Gene 7 (*LOC100274850*) (Figure 2g). Both genes showed exceptionally high constitutive transcript accumulation in the resistant line SMH 57524, followed by a rapid decline after *Fusarium* inoculation and a subsequent shift toward the strongest expression response in SMH 57023. Before inoculation, Gene 14 transcript abundance in SMH 57524 reached approximately 40.6, markedly exceeding the levels recorded in the remaining genotypes. Moderate basal expression was detected in SMH 57324 and SMH 57624, at approximately 5.7 and 4.6, respectively, whereas SMH 57023 and the susceptible cultivar Szyldecka showed comparatively low transcript levels. The initial predominance of SMH 57524 diminished rapidly after inoculation, with expression decreasing to approximately 5.0 at 6 hpi and 3.3 at 12 hpi. At 24 hpi, the expression profile changed considerably, with transcript levels increasing in most genotypes and reaching their highest post-inoculation values. At 24 hpi, the highest transcript abundance was detected in SMH 57023 (approximately 7.3), followed by Szyldecka (approximately 5.6).

Thus, as previously observed for Gene 7, the genotype exhibiting the highest constitutive expression differed from that showing the strongest response following inoculation. By 48 hpi, this response had subsided, and expression decreased to below approximately 2.2 in all genotypes.

#### 2.2.15. Analysis of the Expression of the Candidate Gene Encoding Peroxisomal Carrier Protein LOC100282644

Gene 15 (*LOC100282644)*, encoding a peroxisomal carrier protein (Figure 3g), was characterized by high basal transcript abundance in Szyldecka (~25.0) and, to a lesser extent, in the resistant line SMH 57324 (~22.6). Following inoculation, expression declined sharply across all genotypes, resulting in the temporary loss of the substantial differences observed under control conditions. The most distinctive feature of this gene was the marked increase in transcript levels observed in Szyldecka at 24 hpi (~39.8), when transcript abundance surpassed even the levels detected before inoculation. Although a moderate increase was also apparent in SMH 57324 and, to a lesser extent, in SMH 57023, none of the resistant genotypes displayed transcript levels approaching those observed in the susceptible background. By 48 hpi, expression decreased again in all analyzed lines.

The predominance of Gene 15 activity in Szyldecka, particularly during the later stages of infection, closely resembles the pattern observed for Gene 13 (*LOC100282707*) and supports the notion that this gene may be associated with processes accompanying successful pathogen establishment rather than with resistance-related responses.

#### 2.2.16. Analysis of the Expression of the Candidate Gene Encoding the Uncharacterized Protein LOC103652964

Unlike several previously analyzed candidate genes, Gene 16 (*LOC103652964*) (Figure 3h) did not display pronounced transcriptional changes following inoculation. Instead, transcript abundance fluctuated moderately among genotypes throughout the experiment. Before inoculation, Szyldecka, SMH 57324, and SMH 57524 showed the highest expression levels, with comparable mean values of approximately 1.76, 1.63, and 1.59, respectively. In contrast, basal transcript abundance in SMH 57023 and SMH 57624 was approximately threefold lower. After inoculation, SMH 57524 temporarily had the highest transcript abundance, reaching approximately 2.08 at 6 hpi. This early increase was not sustained, and transcript abundance declined in all genotypes by 12 hpi, with expression remaining below approximately 1.0. At 24 hpi, the expression profile shifted again, with Szyldecka again showing the highest transcript abundance, reaching approximately 1.78. A smaller increase was also evident in SMH 57324, whereas the other resistant lines maintained lower expression levels. By 48 hpi, transcript abundance had declined across all resistant genotypes and remained below approximately 0.7.

Thus, rather than displaying a strong and uniform induction following inoculation, Gene 16 was characterized by modest genotype-dependent fluctuations, with the highest transcript levels observed in SMH 57524 at 6 hpi and in Szyldecka at 24 hpi. Overall, variation in transcript abundance was driven primarily by shifts in relative expression among genotypes rather than by a coordinated induction following pathogen exposure.

#### 2.2.17. Analysis of the Expression of the Candidate Gene Encoding the Uncharacterized Protein LOC103633003

Gene 17 (*LOC103633003*) displayed a pronounced biphasic expression profile (Figure 3i), with high transcript accumulation both before inoculation and at 24 hpi, although different genotypes dominated expression at each stage. Basal expression was particularly high in SMH 57524 and Szyldecka, reaching approximately 45.3 and 37.2, respectively. Substantial transcript abundance was also detected in SMH 57324 and SMH 57023, whereas SMH 57624 remained clearly separated from the other genotypes, with an initial expression level of only approximately 2.3. The high basal transcript accumulation observed across most genotypes diminished rapidly following inoculation. By 6 hpi, transcript levels had fallen sharply across the genotype panel and remained low at 12 hpi. Interestingly, a second wave of mRNA accumulation was found at 24 hpi. In contrast to the basal profile, SMH 57023 displayed the highest transcript abundance, reaching approximately 26.4, closely followed by Szyldecka at approximately 23.2. SMH 57324, SMH 57524, and SMH 57624 showed more moderate increases, with mean values ranging from approximately 3.8 to 7.1. The observed accumulation was transient, as transcript levels declined to near-background values in all genotypes by 48 hpi.

The expression pattern of Gene 17 closely resembled that of Gene 12 (*LOC100277697*), as both genes showed high basal transcript abundance, a marked decline after inoculation, and a subsequent increase at 24 hpi accompanied by a shift in the dominant expressing genotype. However, the second expression wave was considerably stronger in Gene 17, particularly in SMH 57023 and Szyldecka. Notably, Szyldecka maintained high transcript abundance both before inoculation and during the 24-hpi expression peak. The shift from SMH 57524 predominance under control conditions to peak expression in SMH 57023 at 24 hpi further highlights the pronounced temporal and genotype-dependent reorganization of the Gene 17 transcriptional profile.

### 2.3. Metabolomic Analysis and Metabolite Profiling

To determine whether the genotype-dependent transcriptional responses were accompanied by changes at the biochemical level, a comprehensive metabolomic analysis was performed. The concentrations of selected phenolic acids, flavonoids, and carotenoids, together with the activities of key oxidoreductases, were quantified in resistant (Szyldecka) and susceptible (SMH) maize genotypes following *Fusarium* inoculation. This approach enabled the evaluation of both constitutive metabolic differences present before pathogen challenge and inducible responses developing during the early stages of infection.

#### 2.3.1. Phenolic Acids

##### Ferulic Acid

**Figure 4:**
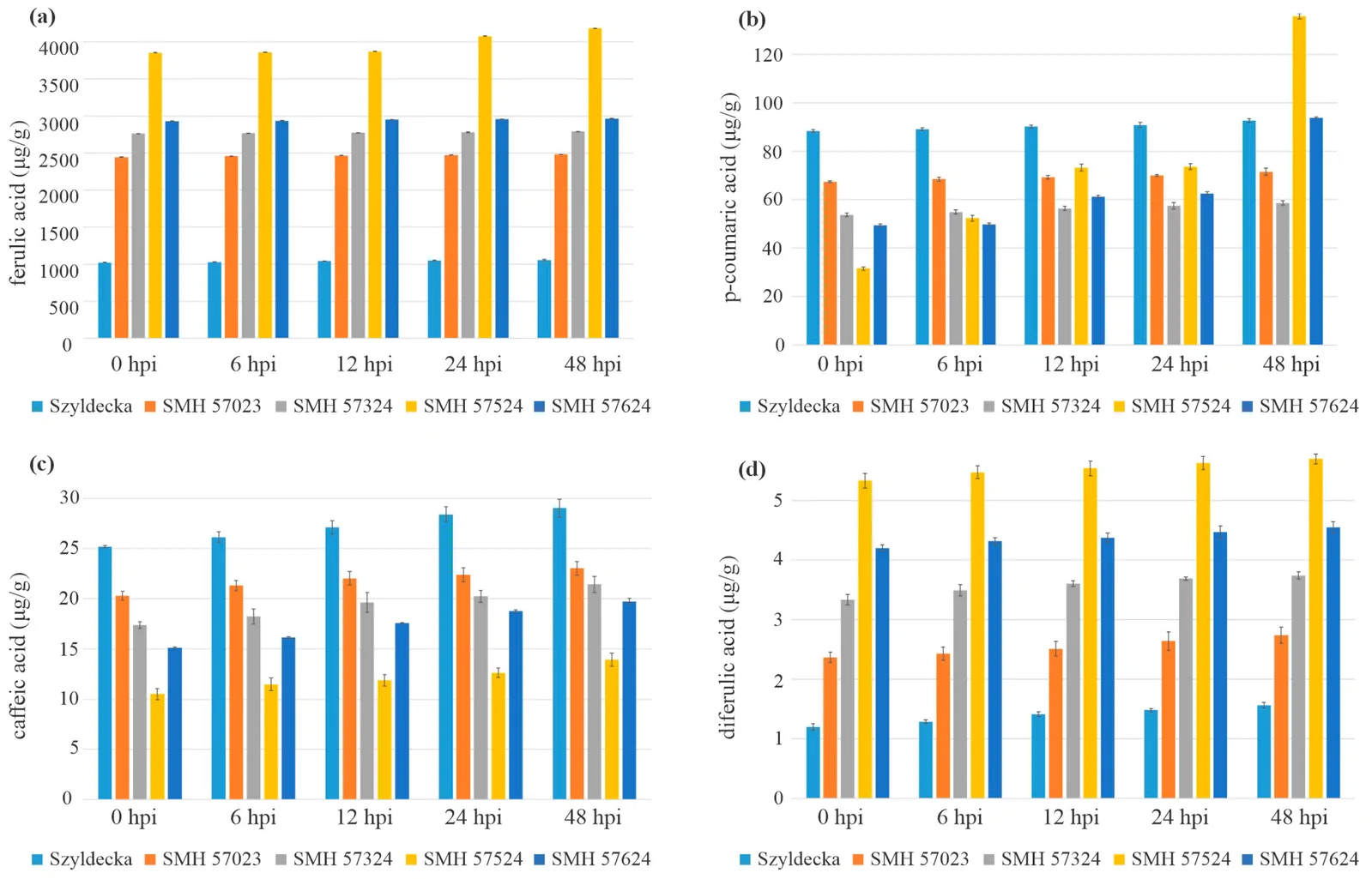
Graphs illustrating the kinetics of changes in phenolic acid content in maize genotypes susceptible and resistant to fungi of the genus *Fusarium*, at selected time points following inoculation. Least significant difference (LSD) values: (**a**) Ferulic acid: Cultivar: 4.458; Time: 4.458; Cultivar × Time: 9.968; (**b**) P-coumaric acid: Cultivar: 1.102; Time: 1.102; Cultivar × Time: 2.464; (**c**) Caffeic acid: Cultivar: 0.738; Time: 0.738; Cultivar × Time: 1.651; (**d**) Diferulic acid: Cultivar: 0.1142; Time: 0.1142; Cultivar × Time: 0.2554.

The concentration of ferulic acid (Figure 4a) in plant tissues remained relatively stable throughout the study period (0–48 hpi), indicating that this metabolite accumulation was largely constitutive. The susceptible cultivar Szyldecka consistently exhibited the lowest ferulic acid concentration among all examined genotypes, and its levels remained constant at all measurement points. In contrast, all four resistant lines maintained substantially higher levels of this compound, although their baseline concentrations differed. SMH 57524 had the highest ferulic acid content, reaching approximately 3850 µg·g^−1^ before inoculation and gradually increasing to more than 4100 µg·g^−1^ at 48 hpi. No rapid changes in ferulic acid concentration were detected following pathogen inoculation. Instead, the most notable difference between resistant and susceptible genotypes was the consistently higher accumulation of this metabolite in the resistant lines, particularly SMH 57524.

##### p-Coumaric Acid

Changes in p-coumaric acid concentration over time revealed clear differences between constitutive and inducible accumulation patterns among the analyzed genotypes (Figure 4b). The susceptible cultivar Szyldecka showed no temporal variation, maintaining a consistently high concentration (approximately 88–93 µg·g^−1^) throughout the experiment. This indicated that inoculation did not stimulate additional accumulation of this metabolite in the susceptible background. The key to resistance is not always the static pool of free acids itself but rather the speed and dynamics of the response induced upon pathogen detection. A susceptible cultivar might accumulate high levels of free phenolic acids because its enzymatic pathways (e.g., specific peroxidases, polyphenol oxidases, or transferases) fail to activate efficiently enough to incorporate these compounds into the cell wall structure or convert them into active phytoalexins. In resistant lines (e.g., SMH 57524 or SMH 57624), these metabolites may be immediately utilized and integrated into cell wall cross-linking processes—a fact supported by the significantly higher levels of diferulic acid and the elevated activity of peroxidases and polyphenol oxidase observed in these lines. Consequently, in resistant lines, a portion of the free monomers may be continuously “consumed” to construct physical barriers, causing their free pools to appear lower, even though the overall metabolic turnover and defense capacity are incomparably higher. The resistant lines displayed more diverse response patterns. SMH 57023 and SMH 57324 exhibited relatively stable p-coumaric acid levels over time. In contrast, SMH 57524 and SMH 57624 showed marked increases in metabolite accumulation. SMH 57524 started from a relatively low concentration (approximately 31 µg g^−1^) and gradually increased to more than 130 µg·g^−1^ at 48 hpi, representing the highest value recorded among the analyzed genotypes. Similarly, SMH 57624 nearly doubled its p-coumaric acid content, reaching approximately 94 µg·g^−1^ by the end of the experiment. Notably, the constitutive concentration of p-coumaric acid in Szyldecka exceeded that of most resistant lines (except SMH 57023), indicating that a high basal level of this metabolite alone was not associated with resistance to *Fusarium*. In contrast, SMH 57524 and SMH 57624 showed the strongest metabolic response, with substantial accumulation during the later stages of infection (24–48 hpi). This increase likely reflects defense-related metabolic adjustments, including cell wall reinforcement and antimicrobial compound synthesis.

##### Caffeic Acid

The susceptible cultivar Szyldecka showed the highest initial concentration of caffeic acid (approximately 25 µg g^−1^), exceeding levels in all resistant lines, which ranged from approximately 10.5 to 20.3 µg g^−1^. As in the case of the p-coumaric acid this observation indicated that high constitutive levels of caffeic acid alone were not sufficient to confer resistance to the pathogen. Following inoculation with *Fusarium*, all analyzed genotypes exhibited a gradual increase in caffeic acid concentration over time. Both the susceptible cultivar and the resistant lines displayed a similar overall accumulation pattern while maintaining the differences observed before inoculation. Consequently, Szyldecka consistently maintained the highest absolute concentration of this metabolite throughout the experiment (Figure 4c).

##### Diferulic Acid

The susceptible cultivar Szyldecka had the lowest diferulic acid concentration throughout the experiment, increasing gradually from approximately 1.2 µg g^−1^ at 0 h to approximately 1.55 µg g^−1^ at 48 hpi. In contrast, the resistant lines consistently maintained higher levels of this metabolite and showed a moderate increase over time. SMH 57023 displayed a gradual rise in diferulic acid concentration from approximately 2.4 µg g^−1^ at 0 h to approximately 2.75 µg g^−1^ at 48 hpi, whereas SMH 57324 increased from approximately 3.35 to 3.75 µg g^−1^ during the same period. Among all analyzed genotypes, SMH 57524 exhibited the highest diferulic acid accumulation, increasing from approximately 5.3 µg g^−1^ before inoculation to approximately 5.7 µg g^−1^ at 48 hpi. Similarly, SMH 57624 increased steadily from approximately 4.2 to 4.55 µg g^−1^.

Despite a gradual increase in diferulic acid concentration across all genotypes, resistant lines consistently maintained substantially higher levels of this metabolite than the susceptible cultivar throughout the experiment (Figure 4d).

#### 2.3.2. Flavonoids

##### Luteolin

**Figure 5:**
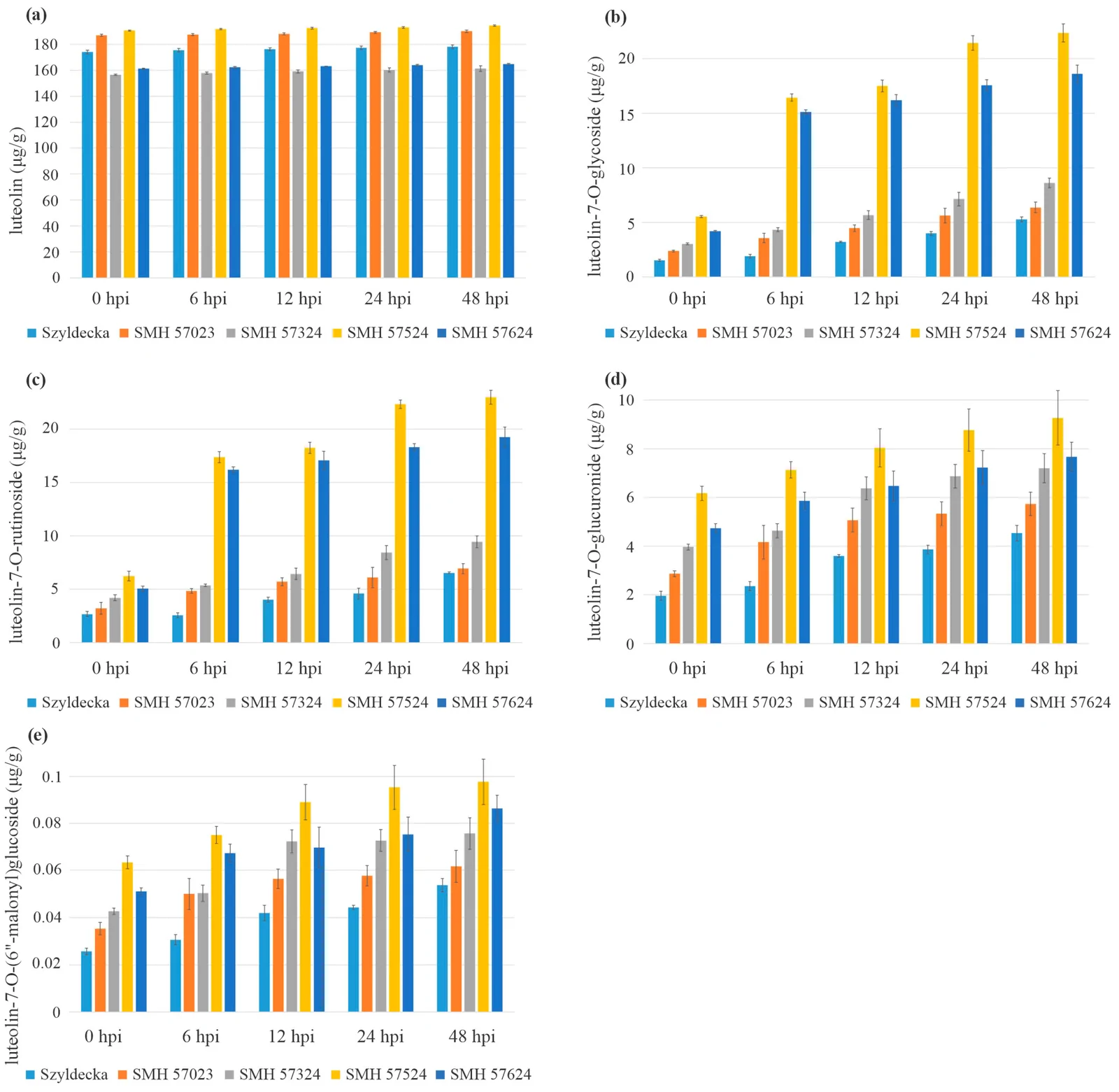
Graphs illustrating the kinetics of changes in flavonoid content in maize genotypes susceptible and resistant to fungi of the genus *Fusarium*, at selected time points following inoculation. Least significant difference (LSD) values: (**a**) Luteolin: Cultivar: 1.245; Time: 1.245; Cultivar × Time: 2.784; (**b**) Luteolin-7-O-glycoside: Cultivar: 0.5476; Time: 0.5476; Cultivar × Time: 1.2245; (**c**) Luteolin-7-O-rutinoside: Cultivar: 0.639; Time: 0.639; Cultivar × Time: 1.428; (**d**) Luteolin-7-O-glucuronide: Cultivar: 0.647; Time: 0.647; Cultivar × Time: 1.447; (**e**) Luteolin-7-O-(6”-malonyl)glucoside: Cultivar: 0.00671; Time: 0.00671; Cultivar × Time: 0.01499.

Prior to inoculation, the susceptible cultivar Szyldecka exhibited an intermediate luteolin concentration (approximately 174 µg g^−1^). The resistant lines showed variable levels of this flavonoid, with SMH 57023 and SMH 57524 exceeding 185–190 µg g^−1^, whereas SMH 57324 and SMH 57624 displayed lower concentrations (approximately 156–161 µg g^−1^). These differences indicated that elevated luteolin content was not consistently associated with resistance. Throughout the experiment, all genotypes showed only minor fluctuations in luteolin concentration, resulting in relatively flat temporal profiles. This pattern suggests that luteolin accumulation was largely constitutive and was not markedly affected by *Fusarium* inoculation during the first 48 hpi (Figure 5a).

##### Luteolin-7-O-Glycoside

The susceptible cultivar Szyldecka exhibited one of the lowest initial concentrations of luteolin-7-O-glycoside (approximately 1.6 µg g^−1^), whereas the resistant lines showed higher baseline levels, ranging from approximately 2.4 to 5.5 µg g^−1^. All genotypes increased metabolite concentration over time, although the magnitude of this response varied considerably. Szyldecka, together with SMH 57023 and SMH 57324, maintained relatively low levels of luteolin-7-O-glycoside throughout the experiment. In contrast, the resistant lines SMH 57524 and SMH 57624 showed a much stronger response, accumulating substantially higher amounts of this flavonoid during infection (Figure 5b).

##### Luteolin-7-O-Rutinoside

A similar pattern was observed for luteolin-7-O-rutinoside (Figure 5c). Prior to inoculation, Szyldecka exhibited the lowest concentration of this flavonoid (approximately 2.7 µg g^−1^), whereas the resistant lines displayed slightly higher baseline levels, ranging from approximately 3.2 to 6.2 µg g^−1^. After inoculation, all genotypes increased metabolite concentration. However, accumulation differed markedly among lines. Only modest changes were detected in Szyldecka, SMH 57023, and SMH 57324, whereas SMH 57524 and SMH 57624 exhibited substantially stronger increases, resulting in the highest luteolin-7-O-rutinoside concentrations at the later stages of the experiment.

##### Luteolin-7-O-Glucuronide

The concentration of luteolin-7-O-glucuronide increased over time in all analyzed genotypes following *Fusarium* inoculation (Figure 5d). Before pathogen challenge, Szyldecka had the lowest level of this metabolite (approximately 2.0 µg g^−1^), whereas the resistant lines showed higher baseline concentrations, ranging from approximately 2.9 to 6.2 µg g^−1^. Although all genotypes accumulated the metabolite, resistant lines consistently maintained higher concentrations than the susceptible cultivar throughout the experiment. This difference was particularly evident in SMH 57524 and SMH 57624, which showed the highest luteolin-7-O-glucuronide levels at successive time points.

##### Luteolin-7-O-(6″-Malonyl)-Glucoside

The accumulation pattern of luteolin-7-O-(6″-malonyl)-glucoside (Figure 5e) closely resembled that observed for the other luteolin derivatives, particularly luteolin-7-O-glucuronide (Figure 5d). Szyldecka exhibited the lowest initial concentration (approximately 0.026 µg g^−1^), whereas the resistant lines displayed higher baseline levels ranging from approximately 0.035 to 0.063 µg g^−1^. Following *Fusarium* inoculation, concentrations increased in all genotypes. However, SMH 57524 and SMH 57624 consistently maintained the highest levels throughout the experiment. These differences further emphasized the greater accumulation of luteolin-derived metabolites in resistant genotypes relative to the susceptible cultivar.

#### 2.3.3. Carotenoids

##### Zeaxanthin

**Figure 6:**
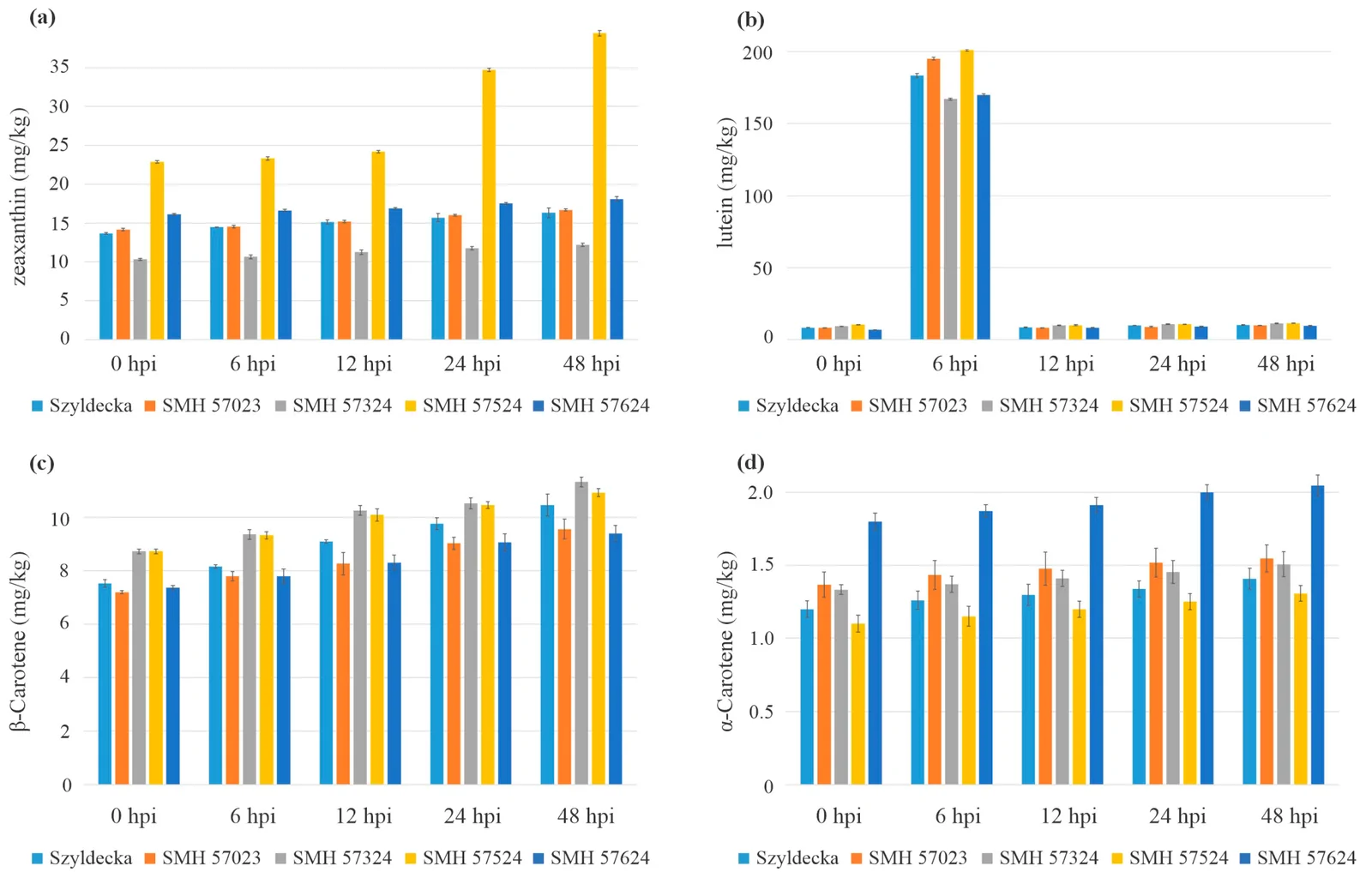
Graphs illustrating the kinetics of changes in carotenoid content in maize genotypes susceptible and resistant to fungi of the genus *Fusarium*, at selected time points following inoculation. Least significant difference (LSD) values: (**a**) Zeaxanthin: Cultivar: 0.3172; Time: 0.3172; Cultivar × Time: 0.7093; (**b**) Lutein: Cultivar: 0.5786; Time: 0.5786; Cultivar × Time: 1.2938; (**c**) α-carotene: Cultivar: 0.2863; Time: 0.2863; Cultivar × Time: 0.6402; (**d**) β-carotene: Cultivar: 0.0891; Time: 0.0891; Cultivar × Time: 0.1992.

The susceptible cultivar Szyldecka exhibited a basal zeaxanthin concentration of approximately 13.8 mg kg^−1^, lower than that observed in most resistant lines, except SMH 57324. In most genotypes, zeaxanthin concentration increased gradually over time following *Fusarium* inoculation. SMH 57524 showed a distinct pattern, with the strongest increase in zeaxanthin accumulation during the later stages of the experiment, particularly between 24 and 48 hpi, resulting in the highest concentration recorded among all analyzed lines. This pattern suggests that zeaxanthin may contribute to resistance-associated responses (Figure 6a).

##### Lutein

Prior to inoculation, and again following 12 hpi, lutein concentrations (Figure 6b) were consistently low and comparable across the susceptible cultivar Szyldecka and the resistant lines. Inoculation with *Fusarium* spp. triggered a rapid but transient increase in lutein accumulation, resulting in a distinct peak at 6 hpi in all analyzed genotypes. Because this response was observed in both susceptible and resistant backgrounds, it was not specifically associated with resistance. Nevertheless, SMH 57524 reached the highest lutein concentration at this time point.

##### β-Carotene

The susceptible cultivar Szyldecka showed an initial β-carotene concentration of approximately 7.5 mg kg^−1^, comparable to the levels observed in SMH 57023 and SMH 57624. Slightly higher baseline concentrations were detected in SMH 57324 and SMH 57524 (approximately 8.7 mg kg^−1^). After *Fusarium* inoculation, all analyzed genotypes showed a gradual, consistent increase in β-carotene concentration over time. The uniform increase in β-carotene accumulation across genotypes suggests that this metabolite may be involved in general stress adaptation processes associated with *Fusarium* infection rather than responses specifically linked to resistance (Figure 6c).

##### α-Carotene

α-carotene accumulation (Figure 6d) increased gradually throughout the experiment in all analyzed genotypes. Before inoculation, Szyldecka contained approximately 1.2 mg kg^−1^ of this metabolite and, except for SMH 57524, had the lowest α-carotene concentration. Both susceptible and resistant backgrounds showed a gradual increase after *Fusarium* inoculation, suggesting that α-carotene accumulation was part of a general response to pathogen-induced stress rather than a resistance-specific process. Throughout the experiment, SMH 57624 maintained the highest α-carotene levels among the analyzed lines, indicating that this metabolite may be involved in genotype-dependent aspects of the response to *Fusarium* infection.

#### 2.3.4. Oxidoreductases

##### Peroxidase

**Figure 7:**
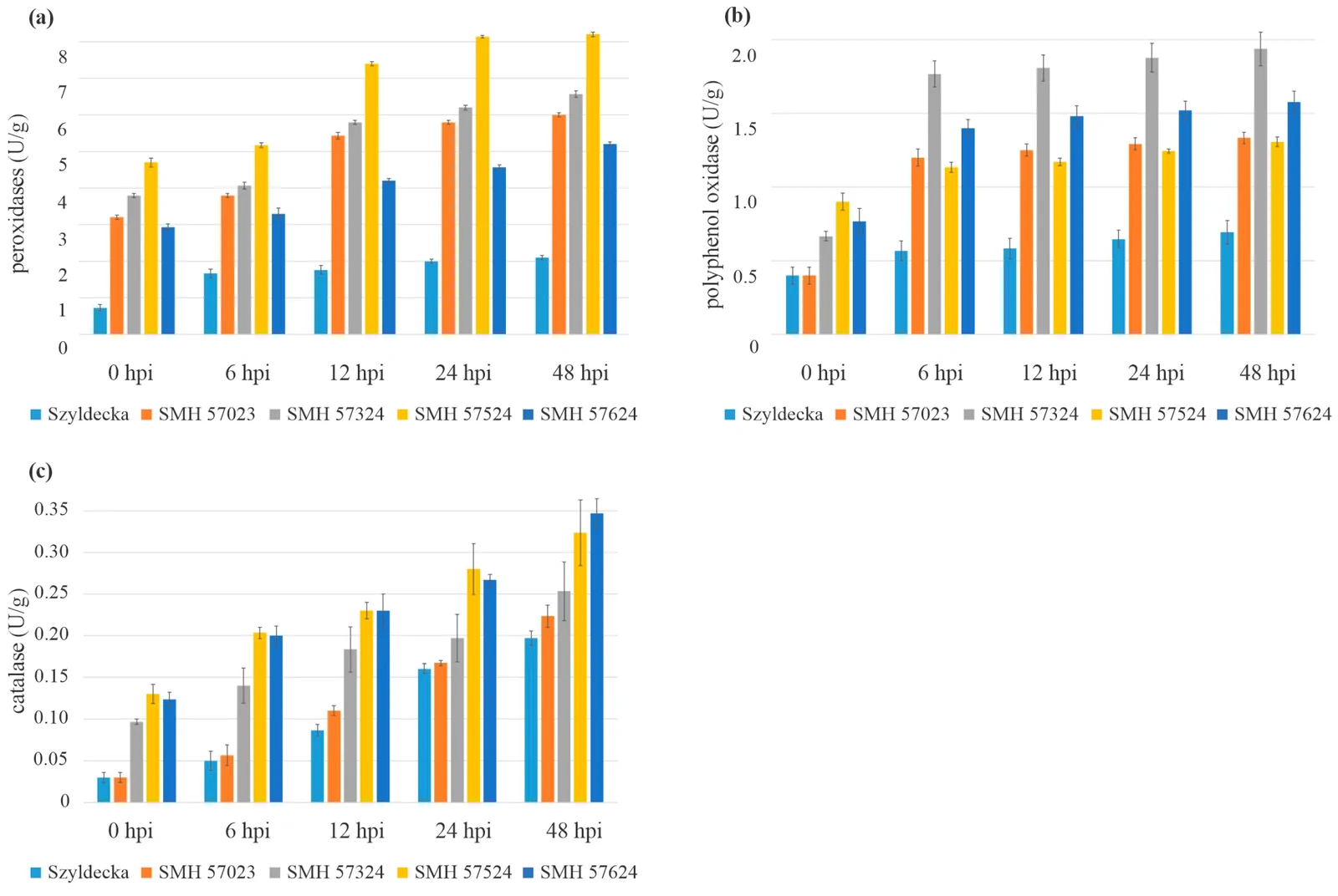
Plots illustrating the kinetics of changes in the levels of oxidoreductases in maize genotypes susceptible and resistant to fungi of the genus *Fusarium*, at selected time points following inoculation. Least significant difference (LSD) values: (**a**) Peroxidase: Cultivar: 0.1009; Time: 0.1009; Cultivar × Time: 0.2257; (**b**) Polyphenol oxidase: Cultivar: 0.0824; Time: 0.0824; Cultivar × Time: 0.1843; (**c**) Catalase: Cultivar: 0.02228; Time: 0.02228; Cultivar × Time: 0.04982.

Peroxidase activity varied considerably among the analyzed genotypes (Figure 7a). Although inoculation increased activity in all lines, resistant genotypes consistently maintained higher values than the susceptible cultivar. Szyldecka displayed the lowest peroxidase activity throughout the experiment, increasing gradually from approximately 0.75 U·g^−1^ at 0 h to approximately 2.1 U·g^−1^ at 48 hpi. In contrast, SMH 57524 and SMH 57324 exhibited the strongest increases following inoculation and maintained activity levels several-fold higher than those observed in Szyldecka. This pattern was consistent with enhanced activation of defense-associated enzymatic responses in resistant genotypes during pathogen challenge.

##### Polyphenol Oxidase

Marked differences in polyphenol oxidase activity were observed among the analyzed genotypes throughout the experiment (Figure 7b). The susceptible cultivar Szyldecka consistently exhibited the lowest activity levels, increasing only slightly from approximately 0.4 U·g^−1^ at 0 h to approximately 0.7 U·g^−1^ at 48 hpi. In contrast, the resistant SMH lines maintained higher activity and showed more pronounced changes following *Fusarium* inoculation. SMH 57324 and SMH 57624 showed the largest increases, reaching substantially higher enzyme activity than the susceptible cultivar. These findings indicate a stronger activation of polyphenol oxidase-associated responses in resistant genotypes during pathogen challenge.

##### Catalase

Catalase activity varied among the analyzed genotypes and increased over time following *Fusarium* inoculation (Figure 7c). Resistant lines generally showed higher basal activity than the susceptible cultivar, particularly SMH 57324, SMH 57524, and SMH 57624, which reached approximately 0.10–0.13 U·g^−1^ prior to pathogen challenge. In contrast, Szyldecka showed one of the lowest initial activity levels (approximately 0.03 U·g^−1^), although catalase activity increased progressively during the course of the experiment, reaching approximately 0.20 U·g^−1^ at 48 hpi. The most pronounced increases were observed in SMH 57624 and SMH 57524, resulting in substantially higher enzyme activity than that recorded in the susceptible cultivar.

### 2.4. Integration of Transcriptomic and Metabolomic Data

**Figure 8:**
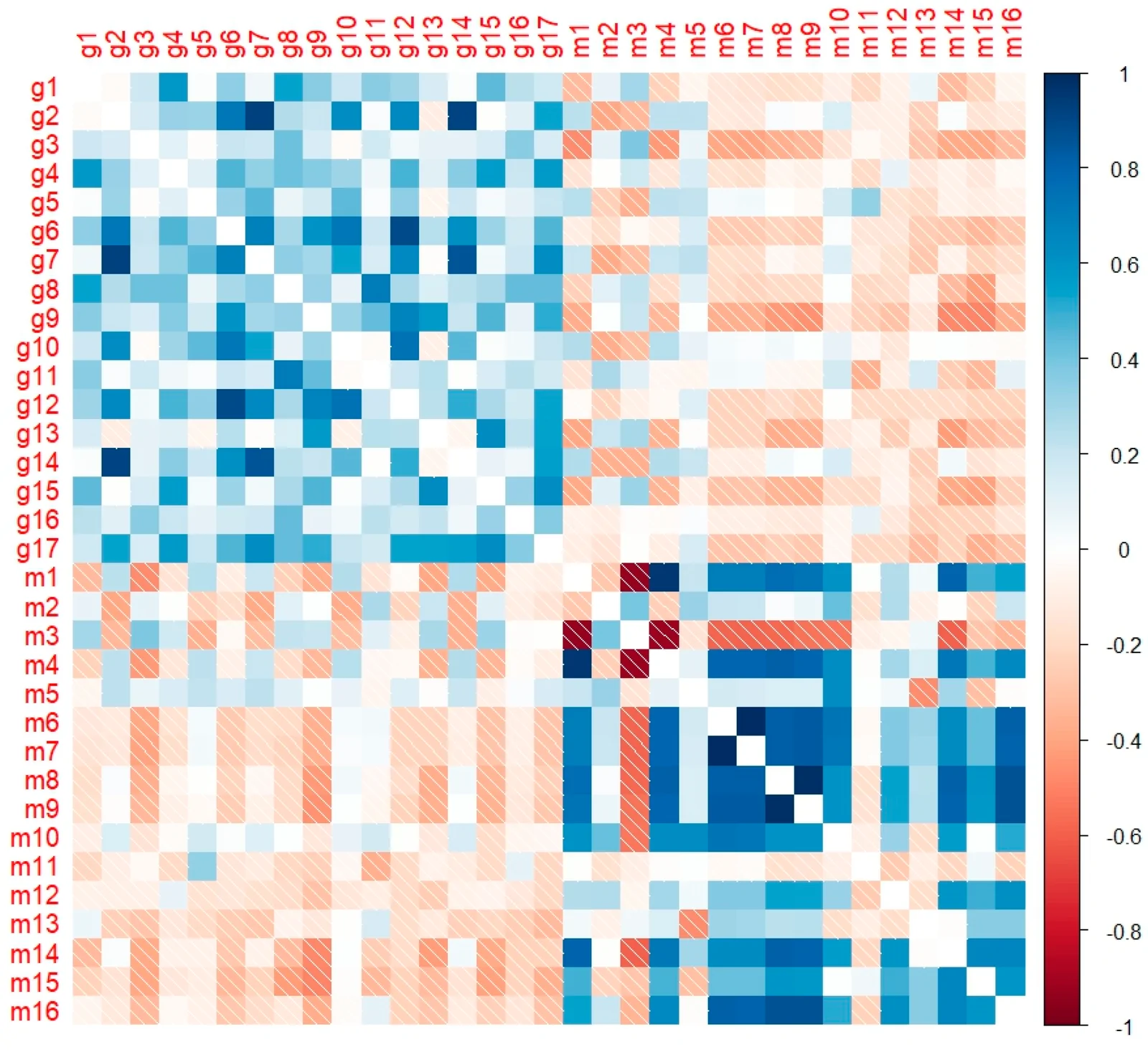
Interdependencies between relative gene expression and metabolite content. g1—*LOC100273960*, g2—*LOC103649226*, g3—*LOC100275570*, g4—*LOC100383808*, g5—*LOC100285378*, g6—*LOC100285600*, g7—*LOC100274850*, g8—*LOC100278673*, g9—*LOC100383775*, g10—*LOC109942573*, g11—*LOC103644288*, g12—*LOC100277697*, g13—*LOC100282707*, g14—*LOC100280235*, g15—*LOC100282644*, g16—*LOC103652964*, g17—*LOC103633003*, m1—ferulic acid, m2—p-coumaric acid, m3—caffeic acid, m4—diferulic acid, m5—Luteolin, m6—Luteolin-7-O-glycoside, m7—Luteolin-7-O-rutinoside, m8—Luteolin-7-O-glucuronide, m9—luteolin-7-O-(6″-malonyl)glucoside, m10—zeaxanthin, m11—Lutein, m12—β-Carotene, m13—α-Carotene, m14—Peroxidases (POD), m15—Polyphenoloxidase (PPO), m16—Catalase (CAT).

The interrelationships between relative gene expression and metabolite content were evaluated using Pearson linear correlation coefficients (Figure 8). The results indicate 153 pairs that are statistically significantly correlated at the 0.05 level, as well as 100 pairs that are significantly negatively correlated (Table S1).

To investigate potential relationships between transcriptional responses and biochemical changes following *Fusarium* inoculation, an integrative analysis combining gene expression and metabolite profiling data was performed. In addition to evaluating the effects of genotype and time on individual traits (Table 1), regression analyses were used to identify candidate genes whose expression levels were associated with the accumulation of specific metabolites (Table 2). This approach enabled the identification of potential links between transcriptional activity and metabolic responses in resistant and susceptible maize genotypes.

Analysis of variance confirmed that genotype, time, and their interaction significantly influenced most analyzed transcripts and biochemical traits (Table 1). The strongest genotype- and time-dependent variation among transcripts was observed for Genes 2 (*LOC103649226*), 4 (*LOC100383808*), 7 (*LOC100274850*), 9 (*LOC100383775*), 13 (*LOC100282707*), and 15 (*LOC100282644*), whereas Gene 16 (*LOC103652964*) did not exhibit significant effects of any tested factor, consistent with the limited variability observed in its expression profile. At the biochemical level, genotype, time, and their interaction significantly influenced several phenylpropanoids, luteolin-derived metabolites, carotenoids, and oxidoreductase activities, indicating marked differences in response dynamics among genotypes.

Regression analyses identified both positive and negative associations between candidate gene expression and the accumulation of specific metabolites or enzyme activities (Table 2). Ferulic and diferulic acids showed highly similar association patterns, as the accumulation of both metabolites was positively correlated with the expression of Genes 2, 5 (*LOC100285378*), 7, 10 (LOC109942573), and 14 (*LOC100280235*). Notably, several of these genes, particularly Genes 2, 7, and 14, also shared similar expression profiles characterized by high constitutive transcript abundance in resistant genotypes and subsequent post-inoculation activation. The presence of Gene 2 in this group, which encodes putrescine hydroxycinnamoyltransferase, supports a potential link between this transcript set and phenylpropanoid-associated metabolic responses. Accumulation of p-coumaric acid was positively associated with expression of Gene 11, encoding cysteine-tRNA ligase CPS1, chloroplastic/mitochondrial-like protein. In the case of caffeic acid, significant positive associations were detected with the expression of Gene 1, encoding a GDSL esterase/lipase, Gene 3 (*LOC100275570*), Gene 13, encoding phosphoribosylaminoimidazole-succinocarboxamide synthase, and Gene 15, encoding a peroxisomal carrier protein. Interestingly, expression of genes 13 and 15 was predominantly associated with the susceptible genotype Szyldecka (Figure 3e,g). The fact that the expression of genes 13 and 15 was primarily associated with the susceptible Szyldecka genotype suggests that the pathogen proliferates freely within the tissues, inducing severe stress and intense metabolic activity in the plant at a later stage (24–48 h). Higher expression levels in the susceptible variety do not signify an effective defense but rather a systemic response to a massive infection—a response that ultimately proves ineffective because the pathogen has already colonized the plant. Within the carotenoid fraction, lutein was positively associated with Gene 5 expression, whereas no significant positive relationships were detected for the remaining carotenoids.

In addition to positive associations, several transcripts also exhibited significant negative relationships with individual metabolites and enzyme activities, indicating that increased expression of some candidate genes coincided with reduced accumulation of specific biochemical components (Table 2). This pattern was particularly evident for Genes 3 (*LOC100275570*) and 9, both of which displayed multiple negative associations with ferulic acid, diferulic acid, luteolin-derived metabolites, and oxidoreductase activities. Notably, these genes also showed expression patterns enriched in the susceptible genotype Szyldecka (Figure 3c and Figure 4a). A similar, although less extensive, trend was observed for Gene 15 (Table 2, Figure 3g). The inverse relationships detected for these transcripts suggest that their elevated expression was associated with reduced accumulation of biochemical traits that were typically enriched in resistant genotypes.

## 3. Discussion

### 3.1. Candidate Gene Expression and Maize Resistance to Fungi of the Genus Fusarium spp.

Infections caused by fungi of the genus *Fusarium* (primarily *F. verticillioides*, *F. graminearum*, *F. culmorum*, and *F. poae*) constitute one of the greatest threats to the productivity and quality of maize (*Zea mays* L.) crops worldwide. Because fully effective and environmentally safe chemical plant-protection methods do not provide sufficient protection against ear and stalk fusariosis, the development of cultivars with enhanced genetic resistance is a key objective of contemporary agronomy and plant breeding. This resistance is quantitative (polygenic) in nature and strongly dependent on genotype–environment interactions. In the present study, a comprehensive, multifaceted analysis was conducted, integrating infection dynamics, the transcriptional activity of 17 selected candidate genes, and metabolomic profiling (phenolic acids, flavonoids, carotenoids, and oxidoreductase enzymes) in genetically diverse maize lines. The results are consistent with the most recent trends in the international scientific literature of the past decade, expanding knowledge of both constitutive and induced defence mechanisms. The findings indicate that maize resistance to *Fusarium* is associated with diverse, genotype- and time-dependent mechanisms of transcriptional regulation, rather than with uniformly higher expression of all analysed genes. This is supported by the marked variation in kernel colonisation: in the susceptible cultivar Szyldecka, *F. poae* and *F. culmorum* predominated, with colonisation by *F. poae* reaching 69–71%, whereas in resistant lines, values for most species ranged from 0 to 2%, with a single exception of 12%.

These observations are consistent with the findings of numerous authors investigating the colonisation of maize tissues by fungi. As reported by Mesterházy et al. [34] and Butrón et al. [20], differences in susceptibility at the kernel level result from the presence of structural barriers and early recognition of pathogen-derived signals. In turn, Ottaviani et al. [35] and Jia et al. [36] emphasise that a critical period for limiting the development of *Fusarium* fungi occurs during the early and middle stages of kernel development (the milk stage), when specific genetic mechanisms are activated. This is also supported by studies by Zhou et al. [37] and Scauflaire et al. [11], which highlight the important role of species specificity within the *Fusarium* complex in relation to the profile of infected host tissues.

Analysis of the expression of the 17 candidate genes revealed higher expression levels for several of them in the susceptible cultivar Szyldecka. Higher expression of certain genes in the susceptible cultivar does not automatically imply that these genes do not participate in resistance. It may reflect an ineffective attempt to activate defence, more severe infection stress, or disturbances in cellular metabolism. The literature on plant–pathogen interactions indicates that resistance effectiveness is determined not only by transcript levels but also by the timing of activation, the function of the encoded proteins, and the coordination of defence pathways. Thus, high expression in a susceptible genotype may indicate a response to a vigorously developing infection rather than effective protection. The inoculation time, 6pm, should also be considered. Measurements at 6 and 12 hpi were taken at midnight and 6am, respectively, whereas measurements at 24 and 48 hpi were again taken at 6pm. Because the circadian rhythm affects gene expression, metabolism, and plant resistance, the decreases observed during the initial period may partly result from night-associated physiological changes.

Among the genes with particularly high expression in the susceptible cultivar Szyldecka at 24 hpi were G1 (*LOC100273960*), G3 (*LOC100275570*), G4 (*LOC100383808*), G6 (*LOC100285600*), G8 (*LOC100278673*), G13 (*LOC100282707*), and G15 (*LOC100282644*). G3 showed early and sustained activation, G4 reached a pronounced peak at 24 hpi, whereas G13 was characterised by very high constitutive expression and a sharp decline after inoculation. G15 reached an expression level of approximately 39.8 at 24 hpi. These profiles may reflect a stress response or processes accompanying infection development but do not demonstrate that these genes directly increase susceptibility. For G13, its involvement in core purine metabolism should additionally be considered, whereas for G15, a possible association with peroxisomal function and lipid metabolism should be taken into account.

A different expression pattern was observed for G2 (*LOC103649226*), G7 (*LOC100274850*), G10 (*LOC109942573*), and G14 (*LOC100280235*), whose high constitutive or induced expression occurred in selected resistant lines. G2, encoding a hydroxycinnamoyl-putrescine transferase, reached an expression level of approximately 55 before inoculation in SMH 57524, whereas in SMH 57023 it increased to approximately 10 at 24 hpi. G7 and G14 also showed high constitutive expression in SMH 57524, whereas G10 was characterised by strong, time-dependent activation in resistant lines. G5 (*LOC100285378*) displayed a mixed profile: high expression in SMH 57524 before inoculation, but an increase in the cultivar Szyldecka at 24 hpi. G9 (*LOC100383775*), G11 (*LOC103644288*), G12 (*LOC100277697*) G16 (*LOC103652964*), and G17 (*LOC103633003*) likewise showed no unambiguous pattern that would allow them to be assigned exclusively to resistance or susceptibility.

These observations are consistent with the results of recent transcriptomic studies. Wang et al. [38], analysing resistant maize lines infected with *Fusarium verticillioides*, showed that key signalling pathways—including those dependent on jasmonic acid and salicylic acid—are activated in a strongly time-dependent manner. Zeng et al. [39] reached similar conclusions when analysing the transcriptomic profiles of the resistant line J1259.

G2 is the most promising candidate because its expression profile is consistent with involvement in phenolamide metabolism, and the literature indicates that hydroxycinnamoyl transferases may participate in plant defence responses. Studies in rice have demonstrated the involvement of some enzymes from this group in resistance to *Magnaporthe oryzae*. However, this does not provide direct confirmation of an analogous function for maize LOC103649226, although previous studies have emphasised the importance of phenylalanine-derived compounds, polyamine metabolism, and lignin biosynthesis in limiting fungal penetration and spread [40,41,42]. The preferential expression of Gene 2 in resistant lines observed in the present study is consistent with these reports. Similarly, the expression of GDSL esterase/lipase (Gene 1) corresponds with the observations of Gao et al. [43] and Shen et al. [44], who suggested that enzymes involved in lipid remodelling and ester hydrolysis form part of the early stress response, although their activation does not necessarily lead to effective pathogen suppression. Recent studies by Sobiech et al. [29] in maize further emphasise that integrating transcriptomic information with phenotypic resistance data may substantially improve the effectiveness of marker-assisted selection (MAS) strategies, particularly for quantitatively inherited traits such as resistance to *Fusarium*.

G7, G10, and G14 are additional candidates; however, because the functions of their encoded proteins are unknown, their roles require validation.

### 3.2. Metabolite Profile and Resistance Mechanisms

The most pronounced metabolic differences concerned phenolic acids, luteolin derivatives, and the activities of enzymes associated with oxidative balance. The results indicate that resistance was associated both with the constitutive accumulation of selected metabolites and with distinct response dynamics after inoculation.

Ferulic and diferulic acids occurred at higher concentrations in resistant lines than in the susceptible cultivar Szyldecka. In the resistant line SMH 57524, the ferulic acid content increased from approximately 3850 to more than 4100 µg·g^−1^, and the diferulic acid content from approximately 5.3 to 5.7 µg·g^−1^. In the cultivar Szyldecka, the concentration of diferulic acid was only approximately 1.2–1.55 µg·g^−1^. These compounds may promote cross-linking of cell-wall components and the formation of a barrier that hinders pathogen penetration into tissues. These observations are consistent with earlier reports by Stuper-Szablewska and Perkowski [45] and Przybylska-Balcerek et al. [46], who demonstrated that phenolic acids reinforce maize cell walls and may limit tissue degradation by cellulolytic and pectinolytic enzymes produced by *Fusarium* species. A different profile was observed for p-coumaric and caffeic acids. Szyldecka maintained a high, relatively stable concentration of p-coumaric acid (approximately 88–93 µg·g^−1^), as well as the highest initial concentration of caffeic acid (approximately 25 µg·g^−1^). This indicates that the content of free phenolic acids alone does not determine resistance. Their subsequent transformations, incorporation into the cell wall, localisation, and bioavailability may be important.

Resistant lines, particularly SMH 57524 and SMH 57624, also exhibited higher concentrations of selected luteolin glycosides. The coordinated increase in luteolin glycosides after inoculation suggests activation of flavonoid metabolism during pathogen infection. This observation is consistent with the results of Förster et al. [47], who showed that *Fusarium* infection may induce the accumulation of flavonoid phytoalexins capable of exerting antifungal activity against economically important maize pathogens, including *F. graminearum* and *F. verticillioides*. These compounds may therefore contribute to antioxidant protection and the response to biotic stress; however, their direct antifungal activity was not unequivocally demonstrated in this experiment. Lutein, in contrast, is a carotenoid synthesised through a distinct isoprenoid pathway. Its associations with candidate-gene expression should be interpreted as potential indirect relationships rather than evidence of the direct involvement of these genes in carotenoid biosynthesis. Lutein showed a rapid but transient increase at 6 hpi in all genotypes, which may be interpreted as a response to the pathogen. However, this increase may also result from circadian rhythm-related effects, as inoculation was performed at 6pm and leaf samples were collected before inoculation, at midnight, at 6am, and at 6pm. In the absence of light, transcription of genes associated with metabolism or photosynthesis may have been physiologically reduced, which could explain the uniform decrease in G1 expression across all genotypes. Nevertheless, the role of carotenoids in the resistance response should not be excluded, because their role as antioxidants and precursors of signalling molecules, including abscisic acid, has been repeatedly emphasised in studies of plant responses to environmental and biotic stress [48,49].

Analysis of oxidoreductases further highlighted biochemical differences between resistant and susceptible genotypes. The activities of peroxidase (POD), polyphenol oxidase (PPO), and catalase (CAT) were consistently higher in resistant lines than in the susceptible cultivar Szyldecka. Peroxidases and polyphenol oxidases participate in cell-wall reinforcement and the generation of antimicrobial quinones, whereas catalase plays an important role in maintaining reactive oxygen species (ROS) homeostasis during infection. Therefore, the increased activity of these enzymes in resistant genotypes is consistent with more efficient regulation of oxidative stress and defence-associated biochemical responses. These observations are consistent with studies by Gill and Tuteja [50] and Sun et al. [42], who identified redox homeostasis as a central component of plant resistance to biotic stress.

### 3.3. The Integration of Transcriptomic and Metabolomic Datasets

The integration of transcriptomic and metabolomic datasets provided additional insight into the biological significance of the observed responses. Notably, the association pattern for ferulic and diferulic acids was highly similar, with their accumulation positively associated with the expression of Genes 2, 5, 7, 10, and 14. Several of these genes, especially Genes 2, 7, and 14, also exhibited closely related transcriptional profiles characterized by elevated constitutive expression in resistant genotypes and inducible activation following inoculation. The recurrence of the same set of transcripts in association with both metabolites suggests a potential link between these genes and phenylpropanoid-related metabolic processes. This observation is especially interesting for Gene 2, which encodes putrescine hydroxycinnamoyltransferase, an enzyme functionally associated with hydroxycinnamate metabolism. Conversely, Genes 3 and 9 displayed multiple negative associations with ferulic acid, diferulic acid, luteolin-derived metabolites, and oxidoreductase activities. Notably, both genes were preferentially expressed in the susceptible genotype Szyldecka. These inverse relationships suggest that increased transcript accumulation of these genes coincided with lower levels of several metabolites and enzymes that accumulated preferentially in resistant lines. A similar, although less pronounced, tendency was observed for Gene 15, supporting the existence of transcriptional programs associated with susceptibility that differ substantially from those linked to resistance. Overall, the observed associations indicate that variation in selected candidate gene expression was linked to changes in metabolite accumulation and enzymatic activity following *Fusarium* inoculation. Although these relationships do not demonstrate direct functional involvement, they identify several transcripts as promising candidates for further investigation of the molecular mechanisms underlying maize resistance and susceptibility [51]. The identified gene–metabolite associations also support the potential use of molecular markers linked to these genes in future breeding programs aimed at improving resistance to *Fusarium* spp.

## 4. Materials and Methods

### 4.1. Plant Material

In the laboratory experiment the plant material comprised 5 reference genotypes, characterized by varying levels of resistance to fungi of the genus *Fusarium*. Of these genotypes, 4 (SMH 57023, SMH 57324, SMH 57524 and SMH 57624) were treated as positive controls and exhibited resistance level 9 to fungi of the genus *Fusarium* (on the 9-point COBORU scale; 1—susceptible, 9—resistant), whilst one genotype (Szyldecka) was treated as a negative control as it exhibited a resistance level of 1. The field experiment involved 20 reference genotypes (ten resistant and ten susceptible to *Fusarium* fungi); kernels were sampled from these for phytopathological analysis to determine the extent of colonization by various *Fusarium* species and inoculum preparation. The ten resistant genotypes included SMH 57023, SMH 57324, SMH 57524, and SMH 57624, while the ten susceptible genotypes included the Szyldecka cultivar. Twenty genotypes were selected for the field analysis to avoid methodological errors. The plant material originated from Hodowla Roślin Smolice sp. z o.o., the IHAR Group (Smolice, Poland) (resistant genotypes) and the Institute of Plant Breeding and Acclimatization of the State Research Institute (IHAR PIB; Radzików, Poland) (susceptible genotype).

These genotypes were selected on the basis of five years’ observations carried out on experimental plots at HR Smolice and at IHAR PIB. Each year, these genotypes were sown in triplicate on 10 m^2^ plots and demonstrated high stability in terms of resistance to fungi of the genus *Fusarium*.

### 4.2. Methodology

#### 4.2.1. Candidate Genes Subjected to Functional Analysis, Associated with Maize Resistance to Fungi of the Genus *Fusarium*

The candidate genes presented in Table 3 were identified by the Team through next-generation sequencing (NGS), association mapping, and physical mapping between 2022 and 2025. The methods and results concerning the identification of candidate genes are described in detail in the publications by Sobiech et al. [52]; Sobiech et al. [29]; and Lenort et al. [53]. In this publication, Table 3 also includes genes that were not reported in the aforementioned publications but proved to be of interest during functional analysis (Table 3).

#### 4.2.2. Growth Conditions and Conduct of the Experiment in the Phytotron and in the Field

A field experiment involving 20 reference maize genotypes was established on 10 m^2^ plots using a randomized complete block design with three replications. Five ears were randomly selected from each plot, and kernels were sampled from them for phytopathological analysis and inoculum preparation.

The experiment involving five maize genotypes was set up under controlled conditions in a phytotron chamber in three technical replicates (15 pots). Seeds were sown into disposable pots with a volume of 2 dm^3^, filled with sterile peat substrate of a specified water-holding capacity and adjusted to a pH of 6.5. The photoperiod was set to 16 h during the day and 8 h at night. The temperature was 24 °C during the day and 18 °C at night. Relative air humidity was maintained at 60–70%. The light intensity was 600 µmol·m^−2^·s^−1^. Watering was carried out systematically, maintaining soil moisture at approximately 70% of field capacity.

The plants were inoculated with a mixture of fungal spores of the genus *Fusarium* at BBCH stage 61 (beginning of flowering). The aerosol was applied using a syringe containing a specified volume of spore suspension directly to the area beneath the leaf sheaths and onto the stigmas. Plant samples for analysis were collected prior to inoculation and 6, 12, 24, and 48 h after inoculation.

#### 4.2.3. Preparation of the Inoculum

Small fragments of infected tissue were excised using a sterile scalpel from ears (collected under field conditions) showing clear symptoms of *Fusarium* infection. To remove bacterial contamination and surface fungi, the fragments were immersed in 70% ethanol for about 3 min, then rinsed three times in distilled water. The dried fragments were placed on Petri dishes containing PDA (Potato Dextrose Agar) medium. Streptomycin was added to the medium to prevent bacterial growth. Once the mycelium had grown, a fragment from the tip of the growing *Fusarium* mycelium was collected using a laboratory loop and inoculated centrally onto a new PDA Petri dish to obtain a pure culture. When a profuse coating (salmon-coloured or pink) appeared on the surface of the colony, 15 mL of sterile distilled water containing 0.01% Tween as a surface-active agent was poured onto the Petri dish. Using a sterile pipette tip, the spores were gently scraped from the surface of the medium. The resulting suspension was filtered through sterile filter paper to separate fragments of mycelium and hyphae from free conidia. A sample of the suspension was then taken, and the spores were counted under a microscope. The suspension was diluted to a concentration of 10^5^ spores·mL^−1^.

#### 4.2.4. Identification of Fungal Species of the Genus *Fusarium*

In the experiment, fungal species of the genus *Fusarium* were identified using macroscopic, microscopic, and molecular methods. Macroscopically, the growth rate of the mycelium, the color of the mycelium on the upper surface (white, pink, salmon, carmine), and the texture (fluffy, cottony, fibrous, or slimy) were assessed. Under the microscope, macroconidia, microconidia and chlamydospores were assessed. For molecular techniques, Sanger sequencing was used. The gene encoding translation elongation factor 1 alpha was analyzed; this is the most important genetic marker for the genus *Fusarium*, enabling the differentiation of almost all species from one another.

#### 4.2.5. RNA Isolation

Total RNA was isolated using the Maxwell^®^ RSC automated system (Promega, Madison, WI, USA) and the Maxwell^®^ RSCsimplyRNA Tissue Kit (cat. no. AS1340) in accordance with the manufacturer’s instructions. Samples were homogenized in lysis buffer supplemented with 1-thioglycerol. The automated purification process included digestion of template DNA with DNase I directly within the cartridge. The isolated RNA was eluted in 30 µL of nuclease-free water. The concentration and quality of the RNA were assessed using a NanoDrop spectrophotometer (Thermo Fisher Scientific, Waltham, MA, USA).

#### 4.2.6. Reverse Transcription

Complementary DNA (cDNA) synthesis was carried out using reagents from Bio-Rad (Hercules, CA, USA). Briefly, 500 ng of total RNA was used for the synthesis. The reverse transcription reaction was carried out in a total volume of 20 µL, using the iScript Reverse Transcription Supermix (Bio-Rad, cat. no. 1708841) in accordance with the protocol supplied with the kit. The reaction was carried out in a thermal cycler according to the following temperature profile: pre-incubation (priming): 5 min at 25 °C, reverse transcription: 35 min at 45 °C, enzyme inactivation: 5 min at 85 °C, and cooling: maintaining the sample at 4 °C. The resulting cDNA products were stored at −20 °C until further analysis by RT-qPCR.

#### 4.2.7. RT-qPCR Analyses

The reaction was carried out in accordance with the manufacturer’s instructions, using a fixed amount of template RNA (200 ng) in a volume of 20 µL. cDNA amplification was performed using the CFX Opus 96 Real-Time PCR System (Bio-Rad Laboratories; cat. no. 12011319). The iTaq Universal SYBR^®^ Green Supermix (Bio-Rad Laboratories; cat. no. 1725120) was used for the reactions. The reactions were carried out in 96-well Hard-Shell PCR Plates (Bio-Rad Laboratories; cat. no. HSP9655) sealed with Microseal ‘B’ PCR Plate Sealing Film (Bio-Rad Laboratories; cat. no. MSB1001). Each measurement was performed in three technical replicates. The thermal conditions for the reactions comprised: an initial denaturation step (30 s at 95 °C) and 40 amplification cycles (10 s at 95 °C and 30 s at 60 °C). Upon completion of the amplification cycles, melting curve analysis was carried out (in the range 65–95 °C in 0.5 °C increments) to confirm the specificity of the reaction products.

Analysis of the raw fluorescence data and determination of the cycle quantification (Cq) values were carried out using CFX Maestro Software (Bio-Rad Laboratories; cat. no. 12013758). The stability of the reference gene expression was verified using the Reference Gene Selector module built into the Bio-Rad CFX Maestro software v. 2.3.

Changes in the expression levels of the target genes relative to the reference genes were calculated using the comparative 2^−ΔΔCq^ method, taking into account the reaction efficiency (E) determined from calibration curves for each of the genes under investigation.

The primers presented in Table 4 were designed to avoid exon-exon boundaries.

#### 4.2.8. Selection and Analysis of Reference Genes

The selection of reference genes began with a literature review aimed at identifying genes exhibiting high expression stability in the biological material under investigation. In this study, a preliminary group of candidates was selected, taking into account genes commonly used in the maize model. Ultimately, three genes were selected: elongation factor 1 alpha (EF1α), beta-tubulin (β-TUB), and cyclophilin (CYP). For each of the selected candidate genes, a standard curve analysis was carried out to determine the reaction efficiency (E) and the coefficient of determination (*R*^2^). These parameters are essential for assessing the technical validity of the qRT-PCR assay prior to proceeding with proper normalization. Based on the standard curves obtained, the genes with the best reaction parameters were selected. Key steps in the procedure included: qRT-PCR analysis on a test cDNA template for the selected candidates. A comparison of the stability of the selected reference genes in test samples using the dedicated Gene Study software on the CFX Maestro system. Finally, the selected genes—beta-tubulin (β-TUB) and cyclophilin (CYP)—were validated to verify their suitability for normalizing data under specific experimental conditions.

#### 4.2.9. Metabolite Analyses

##### Determination of Phenolic Compounds

Phenolic compounds in samples were analyzed after alkaline and acidic hydrolysis [54]. Analysis was performed using an Aquity H class UPLC system equipped with a Waters Acquity PDA detector (Waters, Milford, MA, USA). Chromatographic separation was performed on a Acquity UPLC^®^ BEH C18 column (100 mm × 2.1 mm, particle size 1.7 μm) (Waters, Dublin, Ireland). The elution was carried out gradient using the following mobile phase composition: A: acetonitryl with 0,1% formic acid, B: 1% aqueous formic acid mixture (pH = 2). Concentrations of phenolic compounds were determined using an internal standard at wavelengths λ = 320 nm and 280 nm. Compounds were identified based on a comparison of retention time of the analyzed peak with the retention time of the standard and by adding a specific amount of the standard to the analyzed samples and a repeated analysis. Detection level is 1 µg·g^−1^. Flavonoids (luteolin-7-O-glucuronide, luteolin-7-O-(6″-malonyl)-glucoside, luteolin-7-O-glycoside, luteolin-7-O-rutinoside).

Phenolic acids (p-coumaric acid, caffeic acid, diferulic acid) [55] were analysed.

##### Determination of Carotenoids

Carotenoid isolation and quantification were performed by Acquty UPLC (Waters, Milford, MA, USA) in grain samples by the saponification method. Carotenoids extracts were obtained from ground plant material (0.4 mg) which were triturated with a mixture of acetone and petroleum ether (1:1). Then, after separation of the tested material, the acetone and the hydrophilic fraction were removed from the extract by washing with water. As a result, the ether extract was obtained with a mixture of carotenoid pigments. The extract thus prepared was concentrated in a vacuum evaporator at 35 °C until an oily residue was obtained, then digested in 2 mL of methanol (Merck, Darmstadt, Germany) and subjected to chromatographic analysis. Lutein, zeaxanthin and β-carotene were determined using Acquty UPLC (Waters, Milford, MA, USA) with a Waters Acquity PDA detector (Waters, Milford, MA, USA). Chromatographic separation was performed on a Acquity UPLC^®^ BEH C18 column (100 mm × 2.1 mm, particle size 1.7 μm) (Waters, Dublin, Ireland). Elution was carried out using solvent A-methanol, B-water and tert-butyl methyl ether (TBME). A gradient was applied at a flow of 0.4 mL·min^−1^. The column and samples were thermostated, the column temperature was 30 °C, the test temperature was 10 °C. During the analysis, the solutions were degassed in a Waters device. The injection volume was 10 μL. The registration was carried out at a wavelength λ = 445 nm. Compounds were identified based on spectra in the range from 200 to 600 nm and retention times compared to standards. The following metabolites were analyzed: zeaxanthin, lutein, β-carotene, and α-carotene [56].

##### Determination of Antioxidant Enzyme Activities

The activities of catalase (CAT; EC 1.11.1.6), guaiacol peroxidase (POD; EC 1.11.1.7), and polyphenol oxidase (PPO) were determined spectrophotometrically in plant extracts. Fresh plant material samples (0.5 g) were homogenized on ice in 5 mL of 50 mM potassium phosphate buffer (pH 7.0) containing 1% (w·v^−1^) polyvinylpolypyrrolidone (PVPP). The homogenates were centrifuged at 12,000× *g* for 20 min at 4 °C, and the resulting supernatants were collected for enzyme activity assays. CAT activity was determined according to the spectrophotometric principle described by Aebi (1984) [57], by monitoring the decrease in absorbance at 240 nm resulting from hydrogen peroxide decomposition. POD activity was determined using guaiacol as a substrate, following the method of Chance and Maehly (1955) [58], with modifications. The reaction mixture contained 50 mM phosphate buffer (pH 6.5), 4 mM guaiacol, and 1 mM H_2_O_2_. Enzyme activity was monitored as the increase in absorbance at 470 nm. PPO activity was determined using catechol as a substrate in phosphate buffer (pH 7.0). The oxidation reaction was monitored spectrophotometrically by recording the increase in absorbance at 420 nm. Enzyme activities were calculated from the initial linear rates of absorbance change, corrected for the corresponding blanks, and expressed as U·g^−1^.

#### 4.2.10. Statistical Analysis

The significance of differences in the mean susceptibility of the studied maize genotypes to infection by individual species of the *Fusarium* complex was tested using a *t*-test. The consistency of the empirical distribution with the normal distribution was tested using the Shapiro–Wilk test [59]. The assessment of homogeneity of variances across genotype-determined groups was performed using Bartlett’s test. Repeated-measures analysis of variance was performed to evaluate the effect of varieties at individual time points on the expression of the studied genes and metabolites. The interrelationships between relative gene expression and metabolite content were evaluated using Pearson linear correlation coefficients. Linear regression analysis was used to assess the impact of the expression of individual genes on the levels of the metabolites under consideration. All statistical analyses were performed using the Genstat v. 24.2 statistical software package [60].

## 5. Conclusions

The results indicate that maize resistance to *Fusarium* fungi is determined by the interaction of transcriptional, metabolic, and enzymatic mechanisms whose activity depends on genotype and time post-inoculation. The susceptible cultivar Szyldecka exhibited substantially greater kernel colonisation, particularly by *F. poae* (69–71%), whereas colonisation by most of the analysed species in resistant lines ranged from 0 to 2%, with one exception reaching 12%. The higher expression of certain genes in the susceptible cultivar does not necessarily indicate their direct involvement in increasing susceptibility; rather, it may reflect a more intense but insufficient response to the developing infection. Interpretation of transcriptional dynamics should also account for circadian rhythms, which may have influenced the observed changes in gene expression and metabolism.

Among the 17 genes analysed, G2 (LOC103649226) emerged as a particularly promising candidate. It encodes hydroxycinnamoyl-putrescine transferase, potentially involved in phenolamide metabolism and the production of defence-related hydroxycinnamate derivatives. Genes G7 (*LOC100274850*), G10 (*LOC109942573*), and G14 (*LOC100280235*), also exhibited expression profiles associated with resistant genotypes; however, their functions require further validation. Integration of transcriptomic and metabolomic data revealed positive associations between the expression of G2, G5 (*LOC100285378*), G7, G10, and G14 and the accumulation of ferulic and diferulic acids. In contrast, the expression of G3 (*LOC100275570*) and G9 (*LOC100383775*) was negatively correlated with selected metabolites and the activities of oxidoreductase enzymes. These relationships suggest the existence of distinct regulatory programmes associated with resistance and susceptibility, although they do not establish direct cause-and-effect relationships.

Metabolic and enzymatic mechanisms also appeared to be important components of resistance. Resistant lines exhibited higher concentrations of ferulic and diferulic acids, as well as increased levels of selected luteolin glycosides and higher activities of peroxidase, polyphenol oxidase, and catalase. These findings suggest that cell-wall reinforcement, flavonoid metabolism, and the regulation of reactive oxygen species homeostasis may contribute to restricting pathogen development. At the same time, the maintenance of high levels of certain free phenolic acids in the susceptible cultivar indicates that defence effectiveness depends not only on metabolite concentrations but also on their transformation, localisation, and bioavailability. The transient increase in lutein observed 6 h post-inoculation may reflect an early stress response, although the influence of circadian rhythms cannot be excluded.

In conclusion, the results support a model of resistance to *Fusarium* based on the coordinated regulation of gene expression, the accumulation of phenolic compounds, and the activity of enzymes associated with oxidative homeostasis, rather than on uniformly elevated expression of all defence-related genes. Genes G2, G7, G10, and G14, together with the associated metabolic traits, represent particularly promising candidates for further functional investigations and the development of molecular markers to support the selection of *Fusarium-*resistant maize cultivars. However, confirmation of their significance will require functional studies and validation across a broader range of genotypes and environmental conditions.

## Figures and Tables

**Table 1:** Mean squares from analysis of variance for repeated measurements.

	Source of Variation	Subject Stratum		Subject × Time Stratum		
		Cultivar	Residual	Time	Time × Cultivar	Residual
	d.f.	4	10	4	16	40
	Gene [1] (*LOC100273960*)	43.39 **	5.708	41.317 **	25.085 **	3.663
	Gene [2] (*LOC103649226*)	434.4 ***	10.18	500.88 ***	381.06 ***	15.08
	Gene [3] (*LOC100275570*)	5.3023 *	1.4077	0.4661 ns	0.9607 ns	0.4347
	Gene [4] (*LOC100383808*)	81.97 **	13.34	447.78 ***	150.38 ***	13.59
	Gene [5] (*LOC100285378*)	7.075 ns	2.72	8.208 *	2.724 ns	1.534
	Gene [6] (*LOC100285600*)	159.55 *	33.16	280.96 **	120.98 **	21.52
	Gene [7] (*LOC100274850*)	383.949 ***	6.486	1087.255 ***	495.582 ***	4.941
	Gene [8] (*LOC100278673*)	1.1478 ns	0.8546	1.935 ***	0.3243 ns	0.1547
	Gene [9] (*LOC100383775*)	29.115 **	4.551	41.921 ***	9.53 ***	1.292
	Gene [10] (*LOC109942573*)	67.14 ns	22.23	61.85 ns	75.73 *	16.93
	Gene [11] (*LOC103644288*)	1.25498 *	0.29689	1.92125 ***	0.13101 ns	0.06171
	Gene [12] (*LOC100277697*)	44.54 ns	17.99	283.16 ***	104.97 **	17.34
	Gene [13] (*LOC100282707*)	869.2 ***	32.5	441.96 *	304.77 *	63.96
	Gene [14] (*LOC100280235*)	217.72 *	39.52	266.42 *	156.65 *	31.49
	Gene [15] (*LOC100282644*)	447.68 **	58.13	461.15 ***	193.15 ***	25.52
	Gene [16] (*LOC103652964*)	1.6385 ns	4.8132	2.3444 ns	0.6298 ns	0.6572
	Gene [17] (*LOC103633003*)	291.27 ns	120.28	1445.27 ***	258.33 *	78.58
Phenylpropanoids (µg·g^−1^)	Ferulic acid	1.68 × 10^7^ ***	1.12 × 10^2^	2.37 × 10^4^ ***	1.19 × 10^4^ ***	18.1
	p-Coumaric acid	2453.4091 ***	9.7589	2280.6621 ***	820.4052 ***	0.3831
	Caffeic acid	462.3805 ***	4.1072	32.5451 ***	0.4153 ns	0.2399
	Diferulic acid	38.622425 ***	0.111568	0.324841 ***	0.001561 ns	0.0024
	Luteolin	3297.8845 ***	12.9077	34.9785 ***	0.2827 ns	0.3756
	Luteolin-7-O-glycoside	569.4838 ***	1.5543	179.5071 ***	21.5686 ***	0.3083
	Luteolin-7-O-rutinoside	557.5621 ***	1.6885	172.7611 ***	22.6153 ***	0.5254
	Luteolin-7-O-glucuronide	45.8208 ***	2.5847	21.5145 ***	0.159 ns	0.3272
	Luteolin-7-O-(6″-malonyl) Glucoside	0.0044 ***	0.000285	0.0023 ***	0.00004 ns	0.00003
Carotenoids (mg·kg^−1^)	Zeaxanthin	676.3475 ***	0.4815	73.7742 ***	27.9498 ***	0.1135
	Lutein	1.57 × 10^2^ ***	1.10	9.07 × 10^4^ ***	1.32 × 10^2^ ***	5.03 × 10^−1^
	β-Carotene	9.7238 ***	0.46787	14.13113 ***	0.08413 ns	0.07353
	α-Carotene	1.171 ***	0.0699	0.09365 ***	0.00063 ns	0.000967
Enzymatic activity (U·g^−1^)	Peroxidases (POD)	52.43287 ***	0.0308	18.6242 ***	0.70053 ***	0.01597
	Polyphenol oxidase (PPO)	2.181123 ***	0.046133	1.368727 ***	0.09495 ***	0.00425
	Catalase (CAT)	0.0564313 ***	0.0025533	0.0787013 ***	0.0006722 ns	0.0005

* *p* < 0.05; ** *p* < 0.01; *** *p* < 0.001; ns—not significant; d.f.—the number of degrees of freedom.

**Table 2:** The influence of gene expression on the values of individual metabolites. Green and red shading indicate significant positive and negative associations, respectively.

Parameters			Gene																
			1	2	3	4	5	6	7	8	9	10	11	12	13	14	15	16	17
Phenylpropanoids (µg·g^−1^)	Ferulic acid	Estimate	−87.6	20.47	−464	−17.8	144.3	−11.8	14.7	−358	−133.6	40.3	−280	−3.2	−41.7	27.5	−34.74	−64	−6.73
		*p*-value	0.004	0.022	<0.001	0.181	0.035	0.389	0.025	0.032	<0.001	0.03	0.189	0.836	0.008	0.023	<0.001	0.506	0.385
		Pr	9.5	6.2	21.3	1.1	4.7	x	6.4	4.9	13	5	1	x	13.7	5.6	13.7	x	x
	p-Coumaric acid	Estimate	0.51	−0.734	2.1	−0.022	−3.17	−0.485	−0.654	3.46	0.011	−1.33	11.12	−0.654	0.391	−0.843	0.233	−1.91	−0.218
		*p*-value	0.456	<0.001	0.404	0.94	0.034	0.104	<0.001	0.349	0.99	<0.001	0.015	0.051	0.228	0.001	0.311	0.361	0.197
		Pr	x	15.6	x	x	4.7	2.3	16.5	x	x	13.3	6.5	3.8	1.2	12.3	0.1	x	0.9
	Caffeic acid	Estimate	0.431	−0.1568	2.069	0.1103	−1.171	−0.0235	−0.1356	1.713	0.405	−0.2814	1.16	−0.0622	0.1688	−0.2097	0.1554	−0.064	−0.0004
		*p*-value	0.011	0.001	<0.001	0.13	0.001	0.756	0.001	0.063	0.074	0.005	0.322	0.465	0.047	0.001	0.006	0.903	0.993
		Pr	7.4	12.9	13.5	1.8	11.8	x	14.7	3.3	3	9	x	x	7.1	12	8.6	x	x
	Diferulic acid	Estimate	−0.0972	0.0299	−0.652	−0.0245	0.205	−0.0144	0.0226	−0.425	−0.1836	0.0609	−0.112	−0.007	−0.0578	0.0405	−0.0477	−0.035	−0.011
		*p*-value	0.039	0.037	<0.001	0.227	0.05	0.493	0.047	0.098	0.003	0.031	0.732	0.768	0.011	0.029	0.002	0.811	0.352
		Pr	4.4	5	17.8	0.7	3.9	x	4.8	2.4	10.3	4.9	x	x	12.7	5.1	10.9	x	x
	Luteolin	Estimate	−0.212	0.267	1.03	0.239	1.815	0.247	0.246	2.43	0.021	0.192	−1.3	0.24	−0.071	0.31	−0.119	0.33	0.144
		*p*-value	0.633	0.068	0.526	0.204	0.061	0.203	0.075	0.31	0.972	0.469	0.667	0.272	0.682	0.073	0.423	0.806	0.186
		Pr	x	3.5	x	0.9	3.4	0.9	3.5	0.1	x	x	x	0.3	x	3	x	x	1
	Luteolin-7-O-glycoside	Estimate	−0.258	−0.0758	−2.747	−0.1353	0.135	−0.216	−0.1028	−2.1	−0.915	0.04	0.54	−0.211	−0.169	−0.0705	−0.1883	−0.571	−0.1327
		*p*-value	0.242	0.29	<0.001	0.149	0.784	0.024	0.105	0.077	0.001	0.764	0.719	0.051	0.074	0.418	0.01	0.4	0.014
		Pr	0.5	0.2	14.5	1.5	x	5.5	2.7	2.9	12.1	x	x	3.8	5.3	x	7.5	x	6.8
	Luteolin-7-O-rutinoside	Estimate	−0.283	−0.0824	−2.8	−0.145	0.18	−0.2197	−0.1072	−2.27	−0.911	0.033	0.41	−0.212	−0.1649	−0.079	−0.1822	−0.567	−0.1329
		*p*-value	0.197	0.246	<0.001	0.119	0.711	0.021	0.087	0.054	0.001	0.801	0.785	0.048	0.08	0.361	0.012	0.4	0.013
		Pr	0.9	0.5	15.4	2	x	5.8	3.2	3.7	12.1	x	x	4	5	x	7.1	x	6.9
	Luteolin-7-O-glucuronide	Estimate	−0.1067	0.0051	−0.768	−0.0135	−0.009	−0.0578	−0.007	−0.635	−0.3286	0.0169	−0.188	−0.0549	−0.0854	0.01	−0.0665	−0.228	−0.0341
		*p*-value	0.11	0.81	0.001	0.638	0.95	0.048	0.685	0.078	<0.001	0.675	0.682	0.096	0.006	0.706	0.002	0.268	0.038
		Pr	2.1	x	12.1	x	x	4	x	2.9	17.4	x	x	2.4	14.7	x	10.6	0.3	4.5
	luteolin-7-O-(6″-malonyl)glucoside	Estimate	−0.001	−0.00003	−0.007	−0.0002	−0.0004	−0.0007	−0.0001	−0.006	−0.003	0.00003	−0.002	−0.0007	−0.0008	0.000004	−0.0007	−0.002	−0.0004
		*p*-value	0.131	0.88	0.003	0.469	0.777	0.025	0.415	0.079	<0.001	0.947	0.639	0.044	0.009	0.989	0.002	0.236	0.018
		Pr	1.8	x	10	x	x	5.4	x	2.9	19.2	x	x	4.1	13.6	x	11.2	0.6	6.1
Carotenoids (mg · kg^−1^)	Zeaxanthin	Estimate	−0.175	0.0811	−1.109	−0.0178	0.709	0.0143	0.0698	0.01	−0.332	0.097	2.12	−0.002	−0.128	0.1075	−0.1273	−0.343	−0.0199
		*p*-value	0.434	0.275	0.176	0.852	0.15	0.885	0.317	0.992	0.264	0.469	0.165	0.983	0.258	0.222	0.089	0.618	0.72
		Pr	x	0.3	1.2	x	1.5	x	0	x	0.4	x	1.3	x	0.7	0.7	2.6	x	x
	Lutein	Estimate	−4.15	−0.587	−2.52	−1.713	14.44	−1.24	−0.665	−23.3	−6.31	−0.51	−49.4	−2	−0.534	−0.884	−1.379	5.9	−1.056
		*p*-value	0.07	0.402	0.766	0.079	0.004	0.219	0.311	0.059	0.037	0.713	0.001	0.076	0.527	0.329	0.073	0.403	0.061
		Pr	3.1	x	x	2.9	9.8	0.7	0.1	3.5	4.5	x	12.3	2.9	x	x	3	x	3.4
	β-Carotene	Estimate	−0.0248	−0.0088	−0.108	0.0123	−0.1176	−0.0214	−0.0172	−0.344	−0.1301	−0.0298	−0.233	−0.0333	−0.0372	−0.0102	−0.0071	−0.137	−0.01784
		*p*-value	0.525	0.482	0.449	0.458	0.17	0.211	0.135	0.101	0.01	0.2	0.381	0.081	0.02	0.508	0.588	0.25	0.062
		Pr	x	x	x	x	1.2	0.8	2	2.3	7.4	0.9	x	2.8	10.4	x	x	0.5	3.4
	α-Carotene	Estimate	0.005	−0.006	−0.083	−0.004	−0.036	−0.00889	−0.006	−0.0289	−0.014	−0.0001	0.082	−0.0073	−0.0017	−0.0076	−0.0056	−0.06	−0.006
		*p*-value	0.553	0.039	0.012	0.254	0.073	0.025	0.018	0.562	0.247	0.984	0.189	0.106	0.358	0.033	0.066	0.027	0.005
		Pr	x	4.8	7.1	0.4	3	5.4	7.3	x	0.5	x	1	2.2	x	4.8	3.3	5.3	9
Enzymatic activity (U·g^−1^)	Peroxidases (POD)	Estimate	−0.1838	0.0026	−0.785	−0.0171	−0.087	−0.0679	−0.0122	−0.996	−0.3593	0.0017	−0.976	−0.053	−0.092	0.0105	−0.073	−0.398	−0.0319
		*p*-value	0.004	0.898	<0.001	0.542	0.547	0.017	0.435	0.004	<0.001	0.967	0.027	0.098	0.003	0.684	<0.001	0.046	0.047
		Pr	9.5	x	13.4	x	x	6.3	x	9.6	22.2	x	5.2	2.4	17.4	x	13.8	4.1	4
	Polyphenol oxidase (PPO)	Estimate	−0.031	−0.006	−0.19	−0.0076	−0.03	−0.019	−0.00895	−0.3	−0.0859	−0.0008	−0.3	−0.0156	−0.014	−0.0078	−0.0182	−0.091	−0.01
		*p*-value	0.04	0.188	<0.001	0.246	0.367	0.003	0.022	<0.001	<0.001	0.932	0.003	0.038	0.024	0.196	<0.001	0.051	0.001
		Pr	4.4	1.1	14.7	0.5	x	10	6.8	16.7	23.1	x	10.1	4.5	9.7	0.9	15.8	3.8	12
	Catalase (CAT)	Estimate	−0.001	−0.00101	−0.0299	−0.0004	−0.0043	−0.003	−0.0014	−0.0175	−0.0127	−0.0003	0.0167	−0.0029	−0.0027	−0.001	−0.002	−0.011	−0.0018
		*p*-value	0.652	0.282	0.005	0.752	0.509	0.018	0.072	0.271	<0.001	0.848	0.407	0.041	0.026	0.325	0.035	0.208	0.01
		Pr	x	0.3	9.2	x	x	6.1	3.7	0.3	13.3	x	x	4.3	9.4	x	4.6	0.8	7.4

Pr—Percentage variance accounted for; x—Residual variance exceeds variance of response variate.

**Table 3:** Candidate genes selected by the Team in 2022–2025.

	Candidate Genes	Locus	Chromosomal Localization	Molecular Marker	Marker Type	Publication
1	GDSL esterase/lipase At4g01130 uncharacterized precursor of the protein	*LOC100273960*	Chr. 9	553	SilicoDArT	[52]
2	Putrescine hydroxycinnamoyltransferase	*LOC103649226*	Chr.2	15097	SilicoDArT	[52]
3	Uncharacterized protein	*LOC100275570*	Chr.3	17777	SilicoDArT	[29]
4	Uncharacterized protein	*LOC100383808*	Chr.7	20607	SilicoDArT	[29]
5	Uncharacterized protein	*LOC100285378*	Chr.5	19066	SilicoDAtT	this study
6	Uncharacterized protein	*LOC100285600*	Chr.9	19254	SilicoDArT	this study
7	Uncharacterized protein	*LOC100274850*	Chr.6	20376	SNP	this study
8	Uncharacterized protein	*LOC100278673*	Chr.7	20701	SilicoDArT	this study
9	Uncharacterized protein	*LOC100383775*	Chr.6	21044	SNP	this study
10	Uncharacterized protein	*LOC109942573*	Chr.9	22200	SilicoDArT	this study
11	Cysteine-tRNA ligase CPS1, chloroplastic/mitochondrial-like	*LOC103644288*	Chr.1	27770	SNP	this study
12	Uncharacterized protein	*LOC100277697*	Chr.6	14586781	SilicoDArT	[53]
13	Phosphoribosylaminoimidazole-succinocarboxamide synthase	*LOC100282707*	Chr.6	7049252	SilicoDArT	[53]
14	Uncharacterized protein	*LOC100280235*	Chr.6	2414058	SNP	[53]
15	Peroxisomal carrier protein	*LOC100282644*	Chr.8	4579116	SilicoDArT	[53]
16	Uncharacterized protein	*LOC103652964*	Chr.9	4777510	SNP	[53]
17	Uncharacterized protein	*LOC103633003*	Chr.7	4584918	SNP	[53]

**Table 4:** Sequences of designed primers used for RT-qPCR analyses.

	Candidate Genes	Locus	Forward	Reverse	Product Length	Tm
1	GDSL esterase/lipase At4g01130 precursor uncharacterized precursor of the protein	*LOC100273960*	CCAAAGGAAACAACGGCCAA	GGAGATCTGGCTGACGACTG	155	59
2	Putrescine hydroxycinnamoyltransferase	*LOC103649226*	CTGGATGCGTTCAAGCAGTG	AGGTTGAGGACCACAGATCG	226	59
3	Uncharacterized protein	*LOC100275570*	TATGGAACCAGCCAGGTGGG	CGTGCAATTCCTTGCCGTAG	171	60
4	Uncharacterized protein	*LOC100383808*	GTCTCCTCTCCACCAACACG	GGTCGTTGCCAGCCTCC	155	60
5	Uncharacterized protein	*LOC100285378*	ATGCTTCCTCAAGGGATGCG	TCCCCAAGACAAACGTGACC	137	60
6	Uncharacterized protein	*LOC100285600*	CAGCTCCAGCTCGTCAAGAA	ATGGTTGTTCTCCGCCTCAG	123	60
7	Uncharacterized protein	*LOC100274850*	CAGAGGACACGACGACCAAA	AGGCAGCGAATTTCACAGGA	235	59
8	Uncharacterized protein	*LOC100278673*	TGGTTTCACCTCAGGTTTTTGT	AGGGAACACGAAGCCATCTG	225	58
9	Uncharacterized protein	*LOC100383775*	GCTGTTGAGGCCACCTACAT	TTCAACTTCCAACGGCGGTA	152	60
10	Uncharacterized protein	*LOC109942573*	CTTGAAGCACAGCGATCCAAC	GTTCGTGTCCCCAATTCCTG	178	58
11	Cysteine-tRNA ligase CPS1, chloroplastic/mitochondrial-like	*LOC103644288*	TTCAAGTTATCATGAGGCTTTGC	TCCATGAGGGCGATTCCAAC	180	57
12	Uncharacterized protein	*LOC100277697*	TTCAAAAAGGCGTGTCAGCG	CGTCGATAGAAGCGCTGGAT	210	60
13	Phosphoribosylaminoimidazole-succinocarboxamide synthase	*LOC100282707*	CCTAGAAGCGCAGGAACCAA	CAATGGCAACCCTCACAGGA	232	60
14	Uncharacterized protein	*LOC100280235*	AACATTCAGGAAGCAGGCAG	AGTAATCCACAACCCTGCGG	190	58
15	Peroxisomal carrier protein	*LOC100282644*	CTGTTATCGTGACACAGCCAT	TCCGGAATTATCCTCCGCTG	248	58
16	Uncharacterized protein	*LOC103652964*	CACACGGCTTTGTGGAGGAT	CATGCAGGTACATGCTCGT	181	57
17	Uncharacterized protein	*LOC103633003*	TGCACAGTAGCCAATGCTCT	AGACGAGTGGGGCTAAGGAT	103	59

## Data Availability

The original contributions presented in this study are included in the article/Supplementary Material. Further inquiries can be directed to the corresponding authors.

## Supplementary Materials

The following supporting information can be downloaded at: https://www.mdpi.com/article/10.3390/ijms27219813/s1.
